# Study on the Optimization of Laser Cladding Process Parameters and Overlap Strategies for CuCrZr Copper Alloy Powders

**DOI:** 10.3390/ma19183887

**Published:** 2026-09-11

**Authors:** Jinsu Yu, Duc Anh Le, Chao Zhang, Ji Zhao

**Affiliations:** School of Mechanical Engineering and Automation, Northeastern University, Shenyang 110819, China

**Keywords:** laser cladding, process parameters, equal-area method, overlap ratio

## Abstract

The quality of laser cladding is influenced by a combination of various process parameters, including laser power, powder feed rate, scanning speed, defocus amount, overlap ratio, and the flow rates of the powder feed gas and shielding gas. Taking into account the adjustment characteristics of laser cladding equipment, this paper selects laser power, powder feed rate, and scanning speed as the primary factors of study and systematically analyzes the patterns of their effects on the quality of the cladding layer. Based on the results of preliminary experiments, a single-factor experimental design was formulated, and the experimental results were analyzed according to the established cladding quality evaluation system. By developing an orthogonal experimental design and employing a combination of range analysis and analysis of variance, the optimal combination of process parameters was determined. Subsequently, the overlap ratio and *Z*-axis lift were derived and calculated using the equal-area method, providing a reference for the rapid estimation of multi-pass and multi-layer cladding coating process Windows.

## 1. Introduction

During the laser cladding process, the proper control of process parameters has a significant impact on the formation quality and in-service performance of the cladding layer [1,2,3]. By optimizing key parameters such as laser power, scanning speed, and powder feed rate, it is possible to effectively improve the temperature distribution in the melt pool and reduce temperature gradients and residual stresses. This, in turn, suppresses the formation of cracks and other defects and improves the density and metallurgical bonding quality of the coating [4,5,6,7]. Due to the characteristics of laser cladding process parameters—such as their multivariable, strong coupling, and nonlinearity—their impact on coating microstructure and properties is relatively complex [8]. Consequently, systematic research focused on process parameter optimization has become one of the key research directions in this field [9,10]. In recent years, scholars both domestically and internationally have conducted extensive research on the optimization of laser cladding process parameters and their effects on coating microstructure and properties, achieving a series of significant advancements.

Yin et al. [11] conducted an orthogonal experiment using multi-pass overlapping laser cladding to analyze the effects of laser power, scanning speed, gas flow rate, and overlap ratio on surface flatness, cladding efficiency, and porosity area. Based on an RBF regression fitting model and the multi-objective particle swarm optimization (MOPSO) algorithm, they obtained the optimal combination of process parameters. After optimization, surface flatness improved by 19%, cladding efficiency increased by 26%, and porosity area decreased by 34%. Zhang et al. [12] employed Abaqus finite element simulation and orthogonal experimental design to analyze the effects of laser power, scanning rate, and spot diameter on temperature, residual stress, and hydrogen concentration during the laser cladding of 2.25Cr1Mo steel. Using the temperature at the cladding–substrate interface, maximum residual stress, and maximum hydrogen concentration as evaluation criteria, they obtained the optimal combination of process parameters through a comprehensive weighted scoring method. Liu et al. [13] combined orthogonal experiments with the controlled variable method to analyze the effects of laser power, scanning speed, powder feed rate, and overlap ratio on the quality of Ni60 alloy coatings deposited via laser cladding on the surface of 316L stainless steel. Using crack density, dilution ratio, and forming coefficient as optimization objectives, they established a multiple regression prediction model and obtained the optimal combination of process parameters through a particle swarm optimization algorithm: laser power of 1405 W, scanning speed of 5.7 mm/s, powder feed rate of 0.4 r/min, and overlap ratio of 50%. Liu et al. [14] established a central composite design-based response surface model to evaluate the relationship between process parameters and the aspect ratio of laser-cladded layers on TC4 titanium alloy. Their statistical analysis revealed that the variation in aspect ratio was mainly governed by laser power and powder feeding rate, whereas the influence of scanning speed was comparatively limited. Deng et al. [15] performed an orthogonal experimental investigation to clarify the influence of shielding gas flow rate, laser power, and scanning speed on the geometric characteristics, microhardness, residual TiC particles, and porosity of the coating. Ultimately, the gray correlation analysis method was used to select the process parameter combination with the optimal overall performance. Yao et al. [16] employed the Taguchi-gray correlation method to design a three-factor, four-level orthogonal experiment. Using cladding layer geometric morphology (melt width, melt height, dilution ratio) and porosity as response variables, they analyzed the effects of laser power, scanning speed, and powder feed rate on the quality of Ni60 + 25%WC laser cladding coatings on Q235 steel surfaces. Through gray correlation analysis, they obtained the optimal combination of process parameters. After parameter optimization, the cladding layer width increased by 22%, the dilution ratio decreased by 58%, and the porosity decreased by 7%. This demonstrates that the Taguchi-gray correlation method can be effectively applied to multi-response parameter optimization in this system.

Shi et al. [17] employed a single-variable experimental method to investigate the effects of three process parameters—laser power, scanning speed, and powder feed rate—on the macroscopic dimensions, microstructure, and microhardness of NiCr20-ZrO laser cladding coatings on the surface of 18CrNiMo7-6 gear steel. Using the coating’s macrostructure, microstructural density, and microhardness as evaluation criteria, the optimal process parameters were determined to be: laser power of 700 W, scanning speed of 2 mm/s, and powder feed rate of 11.1 g/min. Under these conditions, the coating formed well, was free of defects, and exhibited good metallurgical bonding with the substrate. Zhang et al. [18] used an orthogonal experimental design to systematically investigate the effects of laser power, scanning speed, and powder feed rate on the dimensional characteristics (melt width, melt height, width-to-height ratio, and dilution ratio) of laser-clad layers on IN718 alloy. Based on this, they focused on analyzing the microstructural characteristics and mechanical properties of the cladding layer interface under optimal process conditions. The results showed that no planar grains were observed at the interface between the cladding layer and the substrate, and the elemental transition was uniform with no macroscopic segregation. The heat-affected zone exhibited grain coarsening and residual MC and M_23_C_6_ carbides. The microhardness of the cladding layer was approximately 280 HV, while that of the substrate was approximately 475 HV. The average shear strength at the interface was 608.87 MPa. Yang et al. [19] conducted single-pass and multi-pass overlapping laser cladding experiments to systematically investigate the effects of laser power, scanning speed, laser lift-off distance, scanning mode, and substrate tilt angle on the morphology, microstructure, and microhardness of three types of powder coatings—316L stainless steel, 903 nickel-based, and 901 iron-based—applied to the surface of steel rails. Combining Ansys numerical simulations to analyze the temperature and stress fields during the cladding process, the researchers ultimately optimized the process parameters for single-pass cladding. Building on this, the study analyzed the effects of scanning mode, coating depth, and powder type on the friction and wear properties of the coatings through reciprocating sliding friction and wear tests and MJP 30A rolling wear tests. Wu et al. [20] studied laser cladding of a Ni60 + 25%WC coating on the surface of U71Mn steel. They first used a Back Propagation (BP) neural network to establish a predictive model relating laser power, scanning speed, powder feed rate, and the geometric characteristics of the cladding layer to the average surface hardness. To address the poor predictive stability of the BP neural network and its tendency to get stuck in local optima, genetic algorithms and particle swarm optimization were used to optimize the BP network’s weights and thresholds, respectively. Hong et al. [21] studied laser cladding of an In718 nickel-based high-temperature alloy coating onto the surface of 40Cr steel. Through orthogonal experimental design, they obtained experimental data corresponding to 25 sets of process parameters and cladding layer quality indicators, and established a Particle Swarm Optimization-Back Propagation (PSO-BP) neural network prediction model. The relative error between the model’s predicted values and the experimental values did not exceed 6%, and the model accurately captured the nonlinear mapping relationship between process parameters and coating quality.

In summary, due to their multivariable, strongly coupled, and nonlinear characteristics, the process parameters of laser cladding have an extremely complex impact on the formation quality and in-service performance of the cladding layer. Although researchers have conducted extensive parameter optimization studies on various material systems and achieved certain results, these studies have primarily focused on steel and nickel-based alloy systems. Research on the process adaptability of laser cladding for highly conductive copper alloy (e.g., CuCrZr) powders remains relatively limited, with a lack of systematic exploration of process windows and multi-objective optimization strategies. Therefore, this study focuses on CuCrZr copper alloy powder and examines three key parameters—laser power, powder feed rate, and scanning speed—combining single-factor experiments with orthogonal experimental design to systematically analyze the influence of each parameter on the geometric morphology, dilution ratio, and shape factor of the cladding layer. Based on these findings, the equal-area method is introduced to theoretically derive the overlap ratio and *Z*-axis lift amount. The aim is to establish a method for optimizing the laser cladding process and controlling multi-layer overlap formation for the CuCrZr alloy system, thereby providing theoretical and experimental support for the stable fabrication of functional surface coatings on highly conductive copper alloys.

## 2. Quality Evaluation Criteria and Experimental Method Design for Laser Cladding

### 2.1. Quality Evaluation Criteria for Cladding Layers

**Figure 1:**
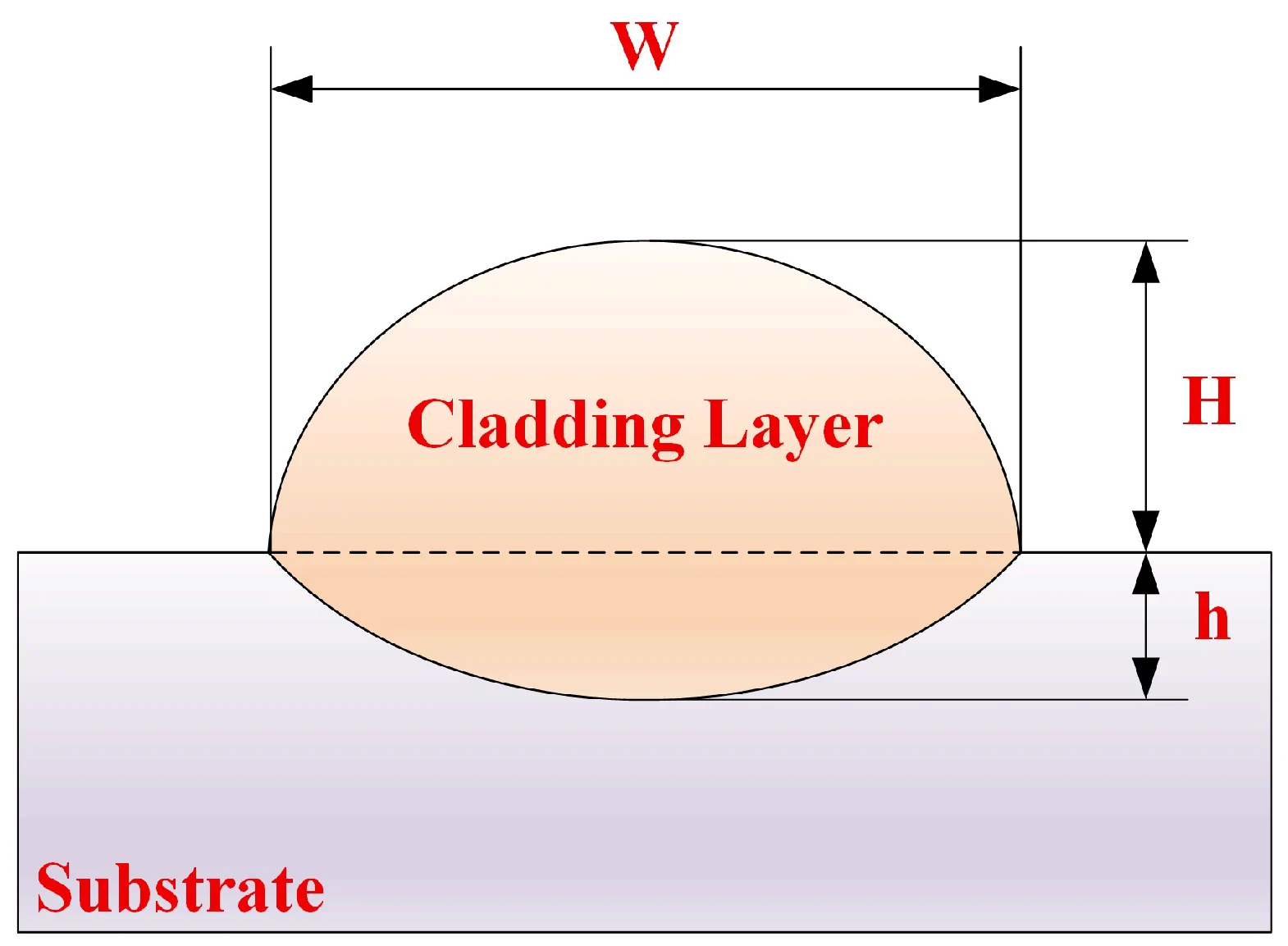
Schematic diagram of the cross-sectional morphology of single-pass cladding.

Typically, during the analysis process, the cross-section of the cladding layer can be approximated as a segment of a circle, as shown in Figure 1. To evaluate the quality of a single-pass cladding layer, an analysis must be based on the morphological parameters obtained from single-pass experiments. In laser cladding processes, the cross-sectional profile of the deposited layer is usually assessed by measuring its width, height, and melt depth, denoted as *W*, *H*, and *h*, respectively. Based on these parameters, comprehensive evaluation indices, such as the shape factor *δ* and the dilution ratio *η* [22,23,24,25], can be further calculated to enable a quantitative assessment of the cladding quality.

The ratio of the cladding layer width W to the cladding layer height *H* is defined as the cladding layer shape factor *δ*. It is generally considered that when *δ* ≤ 2, the cladding layer is more prone to melt pool collapse; when *δ* >> 2, cladding efficiency decreases, which can lead to an increase in the remelted zone and affect the quality of the deposit; whereas when *δ* > 2, better cladding quality can be achieved, and a wider range of overlap ratios is available. The formula for calculating the shape factor *δ* is:

(1) $$\delta \;=\;\frac{W}{H}$$

During laser cladding, the dilution ratio is used to quantify the interaction between the substrate and deposited material, reflecting the extent of substrate melting and its contribution to the final coating composition. The primary objective of laser cladding is to deposit cladding material with specific properties onto the surface of a standard substrate while keeping the dilution ratio as low as possible, so that the resulting coating possesses excellent wear resistance, corrosion resistance, and electrical conductivity—properties that the substrate itself lacks. The dilution ratio is typically calculated using either the compositional method or the area method. This paper employs the area method, and the calculation formula is as follows:

(2) $$\eta \;=\;\frac{S_{2}}{S_{1}+S_{2}}$$

In this equation, *S*_1_ is the cross-sectional area of the cladding layer, and *S*_2_ is the cross-sectional area of the fusion zone. After simplification, the above equation can be expressed as:

(3) $$\eta \;=\;\frac{h}{H+h}$$

In this equation, *H* represents the height of the cladding layer, and *h* represents the depth of the fusion zone. During the cladding process, the formation of a metallurgical bond between the substrate and the cladding material results in dilution of the cladding material. If the dilution ratio is too low, a proper metallurgical bond cannot form between the cladding layer and the substrate; the bond strength will fail to meet service requirements, and the cladding layer may even peel off. If the dilution ratio is too high, the cladding layer absorbs excessive energy, resulting in high thermal stress that can easily lead to cracking, deformation, and collapse. Therefore, the dilution ratio is a key indicator for evaluating the stability of the cladding layer’s chemical composition, microstructure, and mechanical properties. While ensuring a sound metallurgical bond, the dilution ratio should be kept as low as possible to achieve superior performance.

### 2.2. Experimental Equipment and Materials

**Figure 2:**
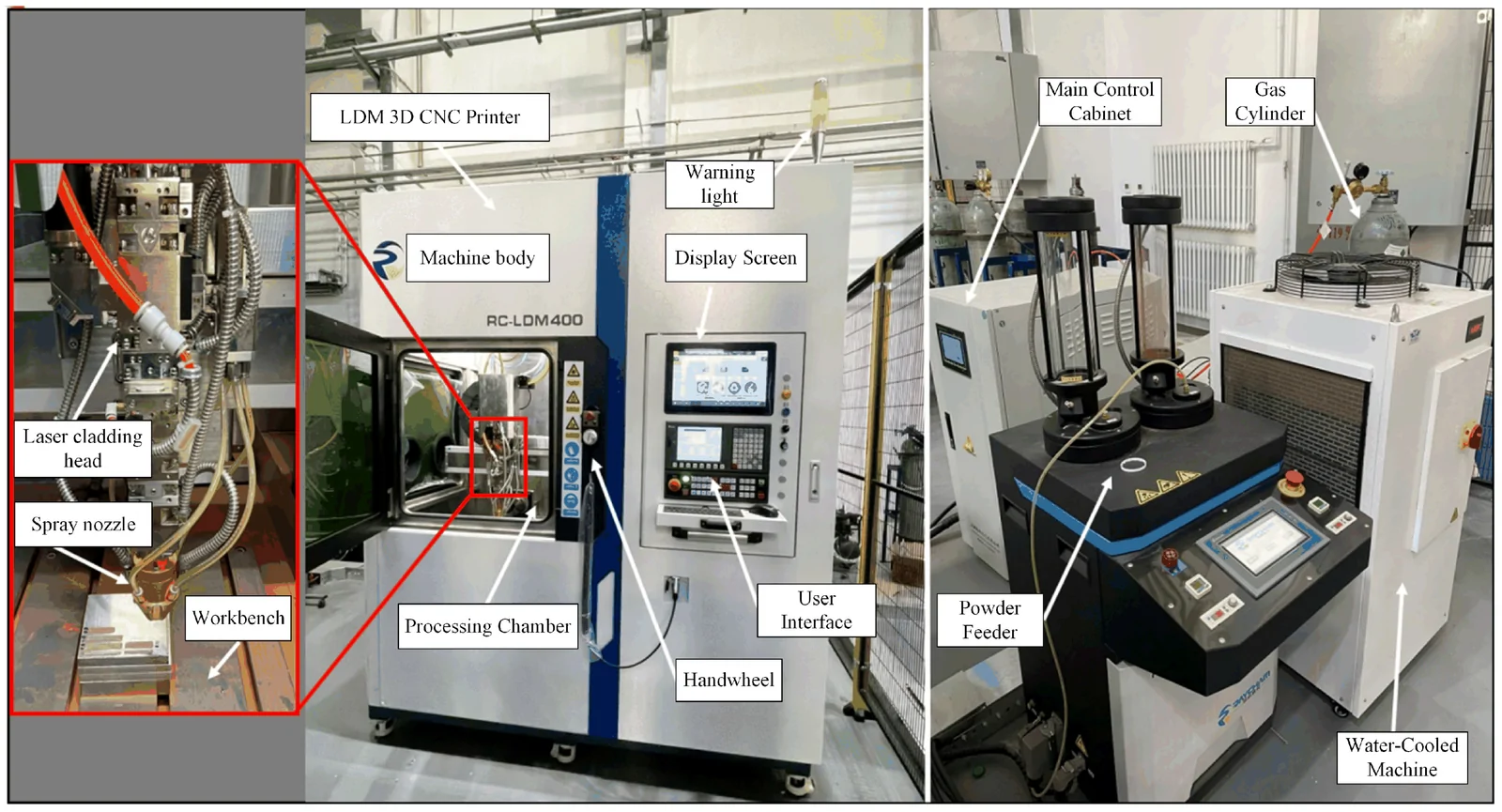
Laser cladding system.

Figure 2 shows the RayCham RC-LDM400 CNC printer and laser cladding system (Nanjing Zhongke Yucheng Laser Technology Co., Ltd., Nanjing City, Jiangsu Province, China) used in the laboratory. The models and technical parameters of the fiber laser used are shown in Table 1. Parameters of powder feeder are shown in Table 2.

**Figure 3:**
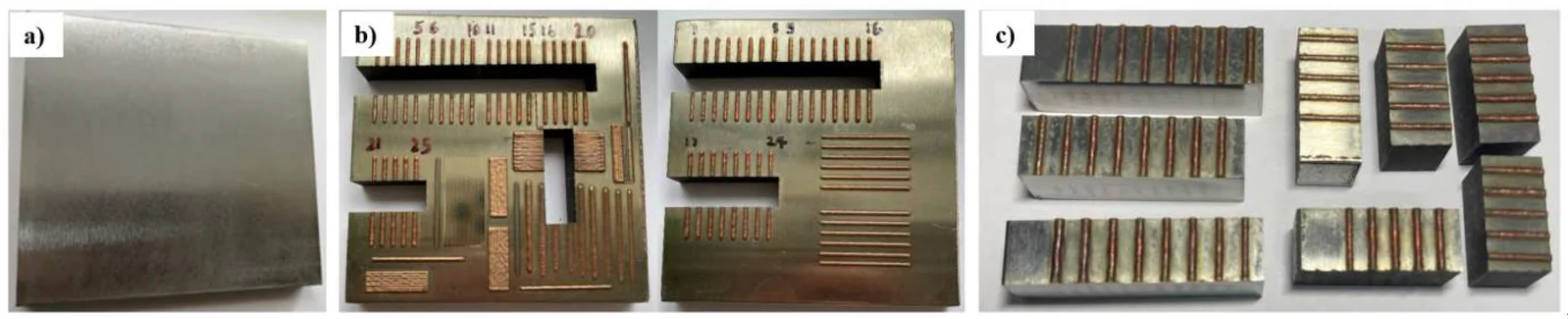
316L Stainless Steel Substrate: (**a**) 316L stainless steel substrate, (**b**) substrate after cutting, (**c**) test specimen.

**Figure 4:**
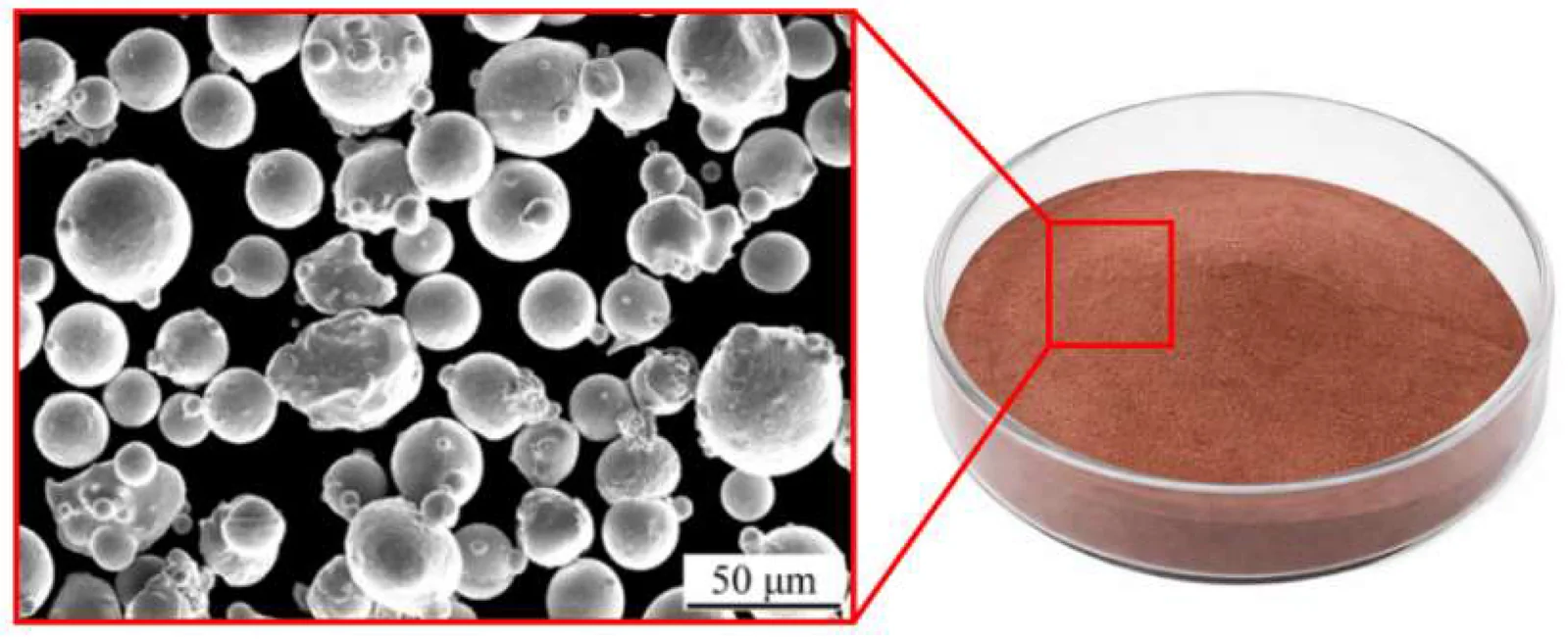
CuCrZr Copper alloy powder.

Figure 3 shows the 316L stainless steel (Shanghai Haocheng Metal Materials Co., Ltd., Shanghai, China) substrate used in the experiment, which measures 100 mm × 100 mm × 10 mm. Table 3 lists its major elements and their concentrations. Figure 4 shows the CuCrZr copper alloy powder (Chengdu Ketailong Co., Ltd., Chengdu City, Sichuan Province, China). Table 4 lists its elemental composition, and its particle size ranges from 35 to 105 µm.

Before conducting laser cladding experiments, it is necessary to first review the relevant theoretical foundations and then complete the preliminary preparations, including equipment installation and commissioning, process parameter setting, and control program development. At the same time, the metal powders used for cladding must undergo pretreatment and performance testing, specifically powder particle size sieving, drying and dehumidification, and assessments of flowability and sphericity, to ensure stable powder feeding and high-quality deposition, thereby laying the groundwork for subsequent experiments.

## 3. A Single-Factor Experimental Study on Laser Cladding

A single-factor experiment was conducted to study the effects of laser power (*P*), powder feed rate (*F*), and scanning speed (*S*), to narrow down the range of process parameters for subsequent experiments. Both the shielding gas and powder feed gas used in the experiment were argon; the powder feed gas flow rate was set to 5 L/min, the shielding gas flow rate to 16 L/min, and the defocus amount to 0. The substrate material was 316L stainless steel, and the cladding material was CuCrZr copper alloy powder. To facilitate subsequent laser cladding, the workpiece should undergo a cleaning process before the experiment. The substrate surface was wiped with alcohol to remove surface oil and prevent contamination of the cladding layer.

### 3.1. Analysis of Single-Factor Experiments on Laser Power

**Figure 5:**
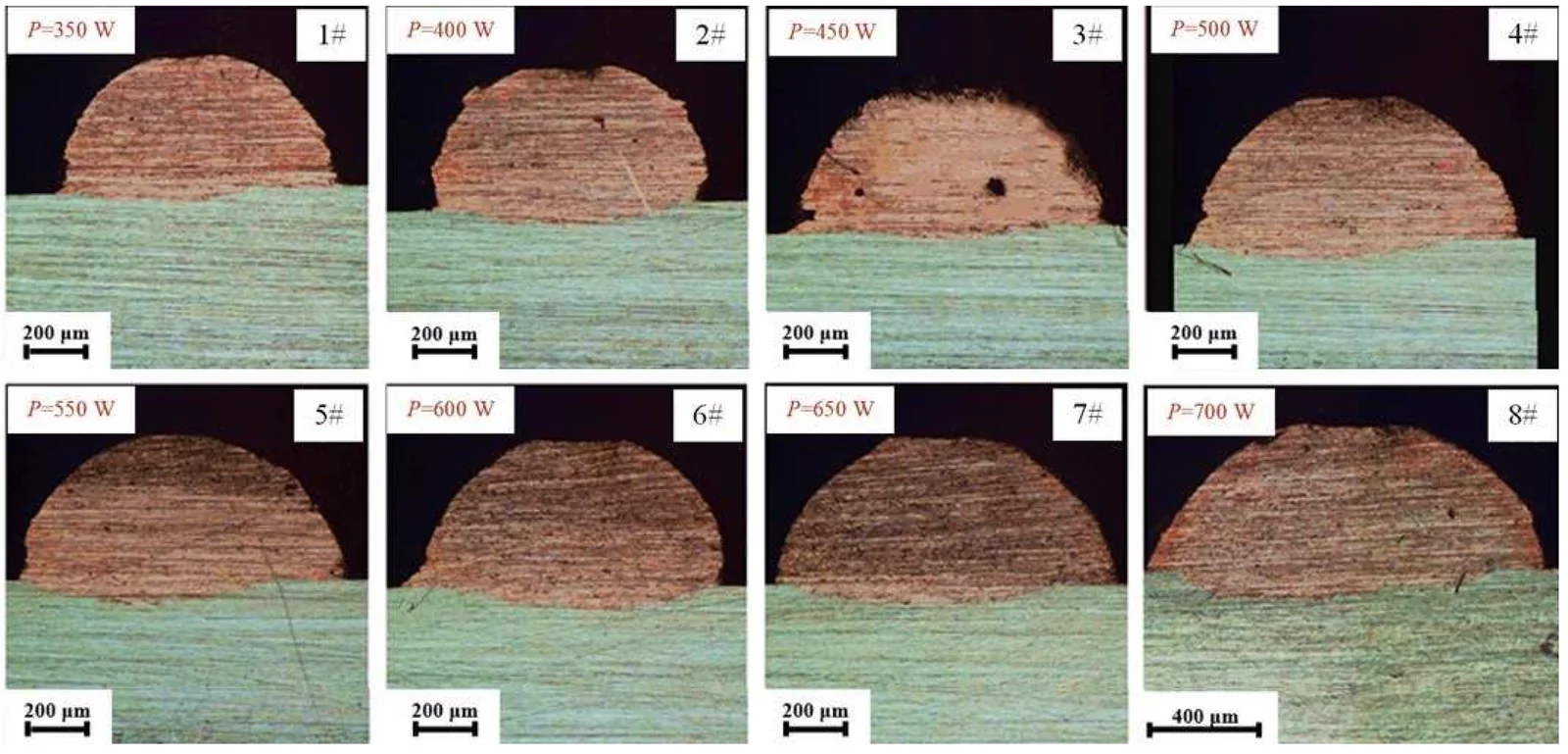
The morphology of the cladding layer in the single factor experiment of laser power.

The laser cladding equipment used in the laboratory has a maximum laser power of 2000 W. However, this single-factor experiment focused only on a narrower power range, with the laser power set between 350 and 700 W. The experimental design and results of the single-factor experiment on laser power are shown in Table 5. The data in the table are the average values of experiments. Figure 5 illustrates the morphology of the cladding layer, which demonstrates good metallurgical bonding between the cladding layer and the substrate.

**Figure 6:**
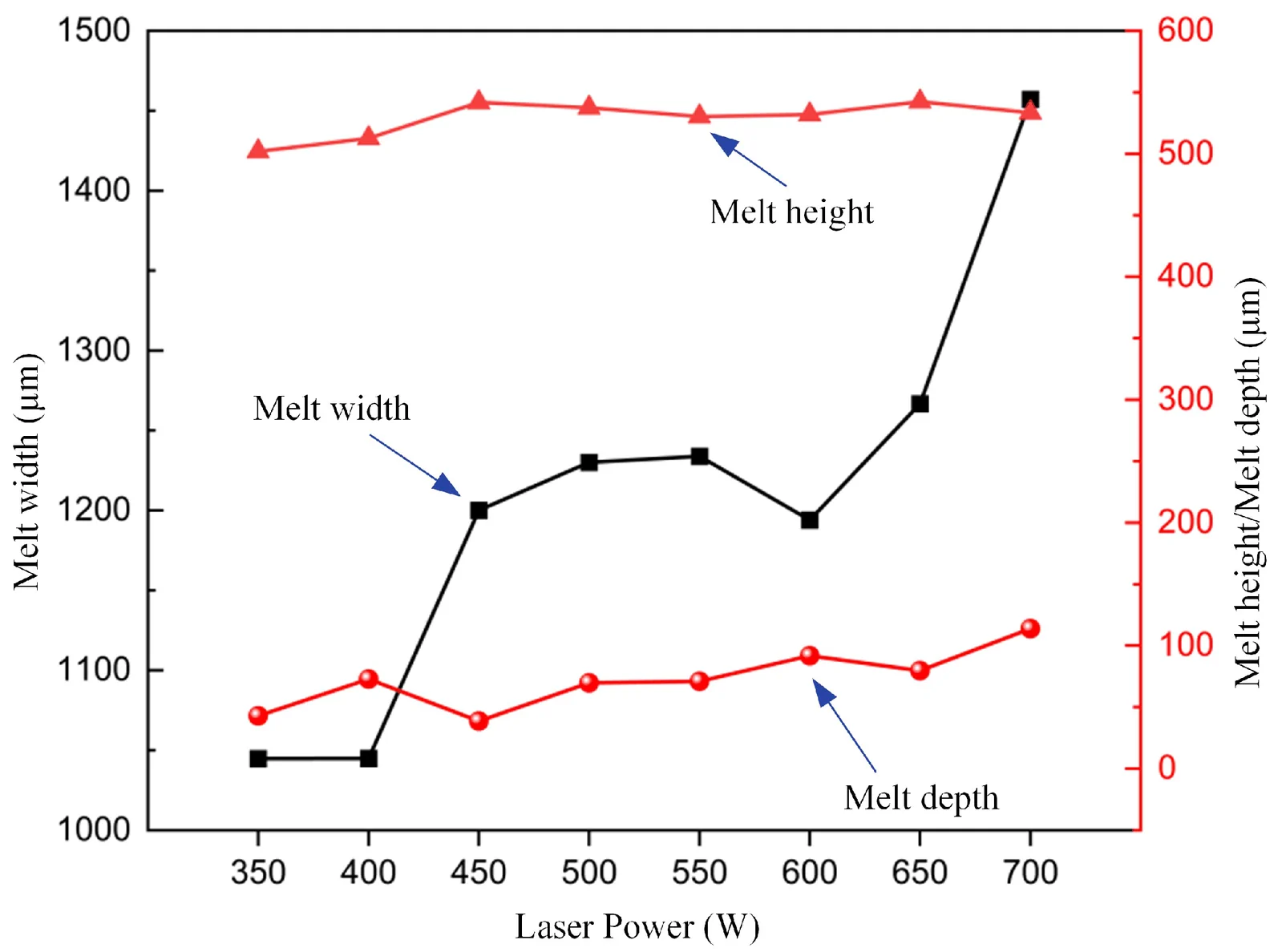
Laser power versus melt width, melt height, and melt depth.

The relationship between laser power and the melt width, melt height, and melt depth is shown in Figure 6. Under conditions of a powder feed rate of 1 r/min and a scanning speed of 660 mm/min, as the laser power increased from 350 W to 700 W, the melt width (*W*), melt height (*H*), and melt depth (*h*) of the cladding layer all changed to varying degrees. Specifically, the melt width (*W*) showed an overall upward trend, increasing from 1044.74 μm to 1457.11 μm. Within the low-power range (350–400 W), changes in melt width were minimal. However, as the power exceeded 450 W, the melt width increased significantly. A slight fluctuation occurred around 600 W, followed by a further significant increase at 700 W. The change in melt height (*H*) was relatively gradual, generally ranging from 502.0 to 542.4 μm. It initially increased with rising power, reaching a high value of 541.9 μm at 450 W, followed by slight fluctuations before stabilizing in the high-power range. The melt depth (*h*) exhibits a fluctuating upward trend as laser power increases, rising from 42.95 μm to 113.84 μm. Fluctuations are more pronounced in the low-power range, while it continues to increase overall above 600 W, corresponding to a gradual deepening of the substrate materials’ melting.

The primary reason for these changes is that increased laser power boosts the input energy, directly raising the temperature of the melt pool and expanding the heat-affected zone. As more powder material is fully melted and spreads to both sides, this directly drives an increase in the melt width; simultaneously, the effect of heat conduction intensifies, with the substrate absorbing more heat, which promotes an increase in melt depth. Due to the combined influence of convection behavior within the melt pool and the substrate’s own thermal conductivity, all forming parameters exhibit some fluctuations in the medium-power range.

**Figure 7:**
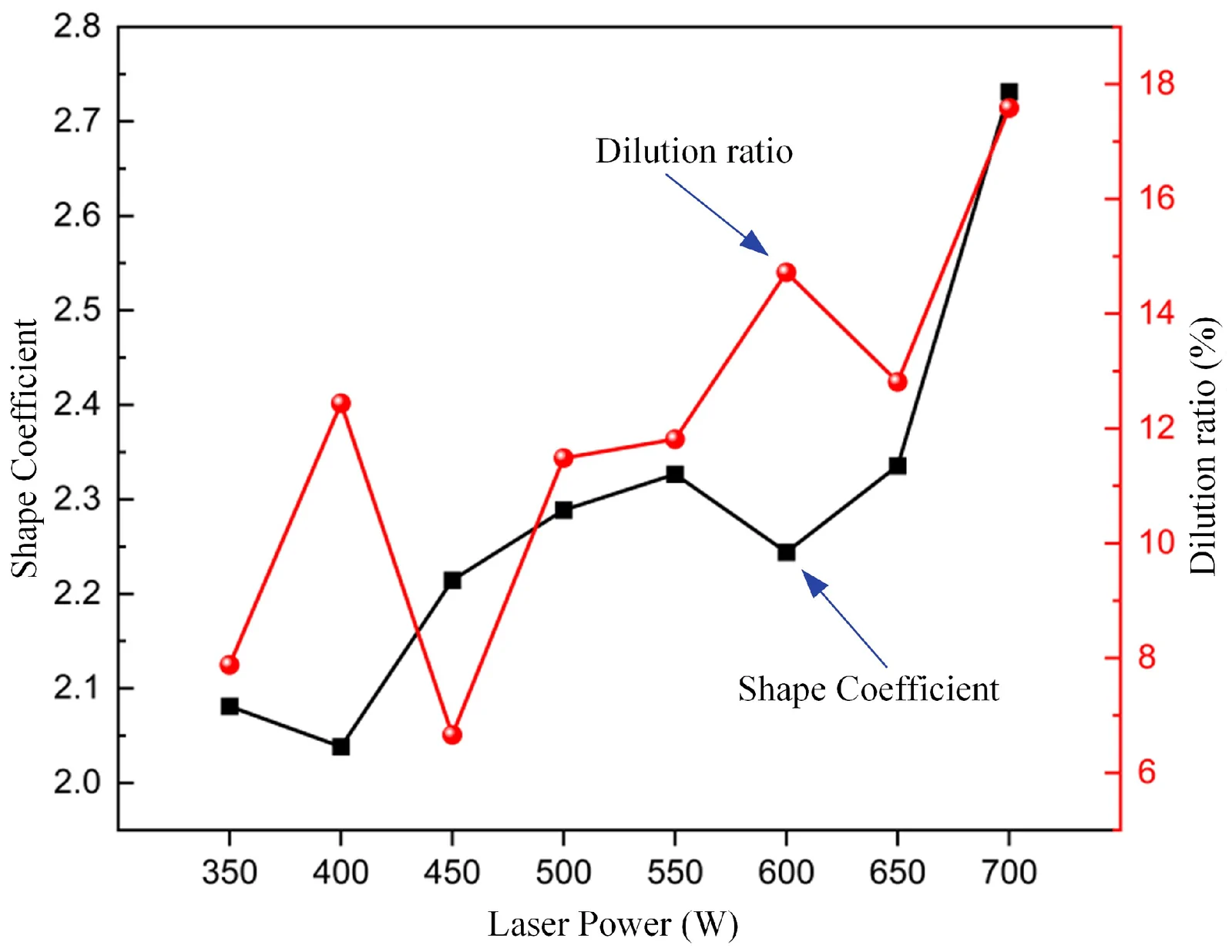
Laser power versus shape factor and dilution rate.

The relationship between laser power, the shape factor, and the dilution ratio is shown in Figure 7. It can be seen that as the laser power increases from 350 W to 700 W, the shape factor remains stable at around 2, and the cladding layer exhibits good morphology. Within the tested dilution range (7.88–17.59%), the cladding layers exhibited continuous cross-sectional profiles without macroscopic interfacial cracks or spallation. Metallurgical bonding quality cannot be fully assessed by dilution ratio alone; direct evidence from interfacial microstructural characterization using scanning electron microscopy/energy dispersive spectroscopy (SEM/EDS, Japan Electron Optics Laboratory Co., Ltd., Tokyo, Japan) and mechanical testing (shear or tensile tests) is required for a definitive evaluation. In this study, the dilution ratio is used as a process indicator rather than a direct measure of bond strength. The *η* > 8% criterion is proposed as a conservative empirical guide based on the observed defect-free macroscopic interface, and future work should incorporate quantitative bond strength testing to validate this threshold.

**Figure 8:**
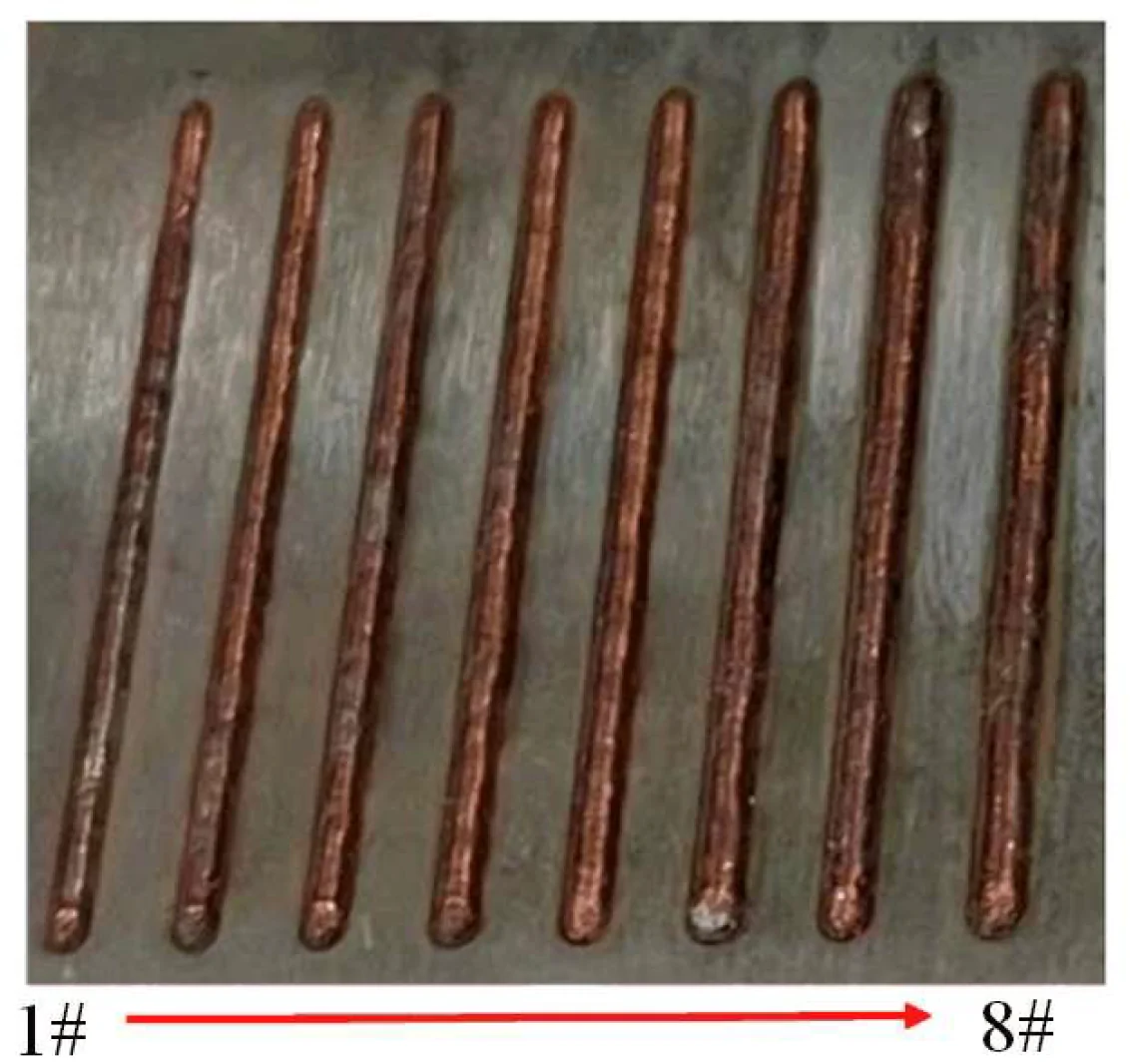
The macroscopic morphology in the single factor experiment of laser power.

The surface morphology of the cladding layer is shown in Figure 8. The surface of the cladding layer is generally smooth and continuous, without obvious defects such as powder adhesion or cracking, and the forming quality is good. When the laser power exceeds 550 W, the local color of the cladding layer becomes darker, and the excessively high power of the surface light will accelerate the oxidation of the cladding layer.

Taking into account that the dilution ratio should be low while ensuring good metallurgical bonding, and that the shape factor should be slightly greater than 2, a laser power of 400–500 W was selected for the orthogonal experiments.

### 3.2. Analysis of Single-Factor Experiments on Powder Feed Rate

**Figure 9:**
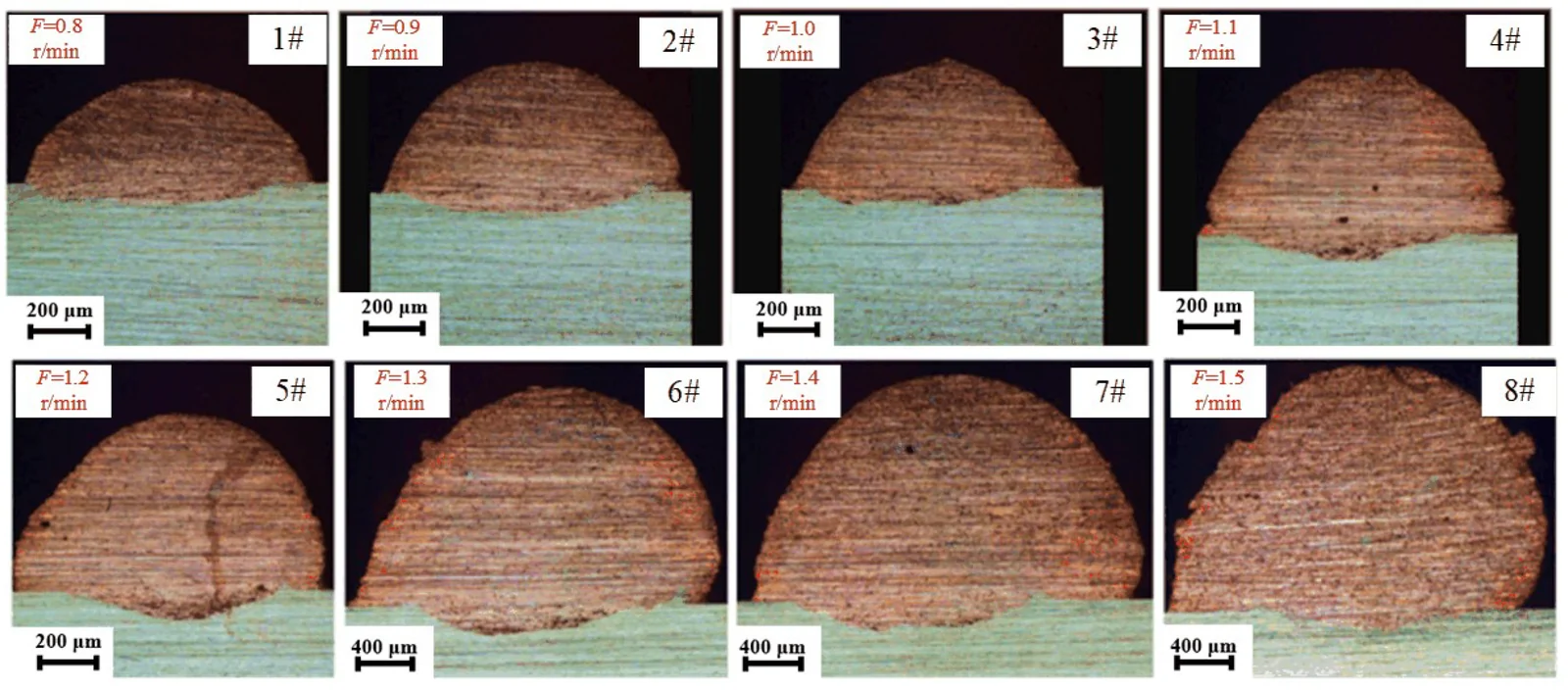
The morphology of the cladding layer in the single factor experiment of powder feed rate.

Based on preliminary experiments, the experimental design and results for the single-factor study on powder feed rate are shown in Table 6. Figure 9 presents the optical microscopy image of the cross-sectional interface. A continuous bonding line is observable, with no macroscopic cracks or voids detected at the interface. This indicates that the cladding layer and substrate are in good contact.

**Figure 10:**
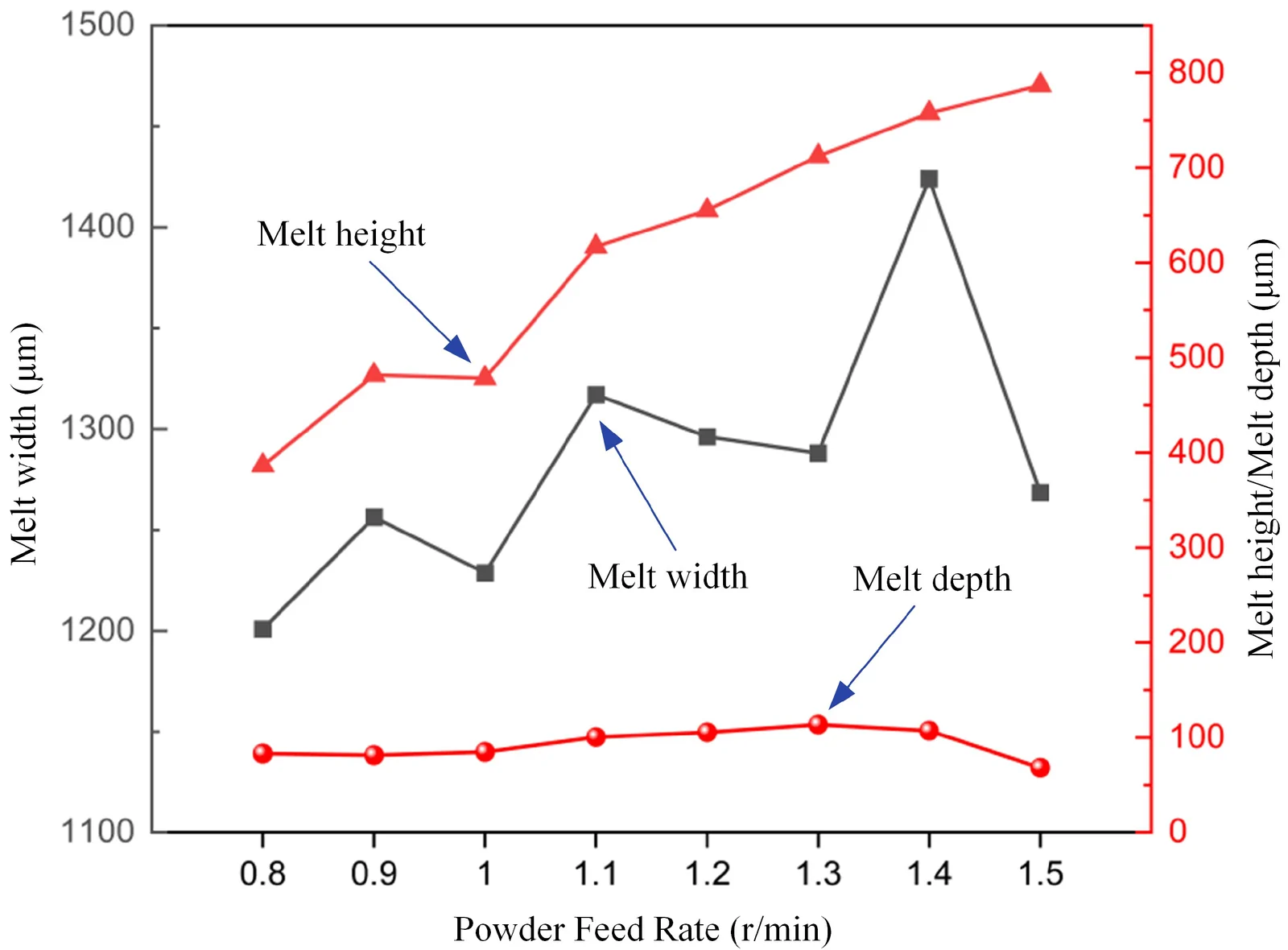
Powder feed rate versus melt width, melt height, and melt depth.

The relationship between the powder feed rate and the melt width (*W*), melt height (*H*), and melt depth (*h*) is shown in Figure 10. Under conditions of 500 W laser power and a scanning speed of 660 mm/min, as the powder feed rate increases from 0.8 r/min to 1.5 r/min, the melt width (*W*), melt height (*H*), and melt depth (*h*) of the cladding layer all change to varying degrees.

In particular, the melt width (*W*) exhibited a generally fluctuating trend, with values ranging from 1200.89 to 1423.93 μm. When the powder feed rate was in the range of 0.8–1.1 r/min, the variation in melt width was relatively gradual. As the powder feed rate increased to 1.2 r/min or higher, the amplitude of melt width fluctuations increased, reaching a maximum of 1423.93 μm at 1.4 r/min, before decreasing slightly at 1.5 r/min.

The melt height (*H*) showed a clear upward trend as the powder feed rate increased, rising from 386.43 μm to 787.10 μm. The rate of increase in melt height was relatively rapid in the low powder feed rate range. After 1.1 r/min, the rate of increase slowed slightly, and it gradually stabilized in the high powder feed rate range. The melt depth (*h*) also exhibited fluctuations as the powder feed rate increased, generally increasing first and then decreasing, with the value changing from 83.18 μm to 68.30 μm. When the powder feed rate was in the range of 1.0–1.3 r/min, the melt depth increased significantly, reaching a maximum of 113.84 μm at 1.3 r/min. As the powder feed rate increased further, the melt depth decreased significantly.

The melting width and melting height generally show a certain positive correlation with the increase in powder feeding rate. The mechanism behind these changes can be explained as follows: when the powder feed rate increases, the amount of powder entering the melt pool per unit time increases, and more laser energy is allocated to the powder melting process. The molten powder accumulates on the substrate surface, directly leading to an increase in melt height. At the same time, the energy transferred to the substrate relatively decreases, inhibiting substrate melting and ultimately resulting in a decrease in melt depth. The melt width is jointly constrained by the laser energy distribution and the thermal flow conditions within the melt pool; therefore, it fluctuates only within a fixed range at different powder feed rates. The coupled effects of convection and heat conduction within the melt pool can cause slight fluctuations in melt pool morphology parameters in the moderate powder feed rate range. Since this experiment did not include real-time observation of the internal flow field of the melt pool, future studies could employ high-speed imaging techniques to further clarify the specific causes of these fluctuations.

**Figure 11:**
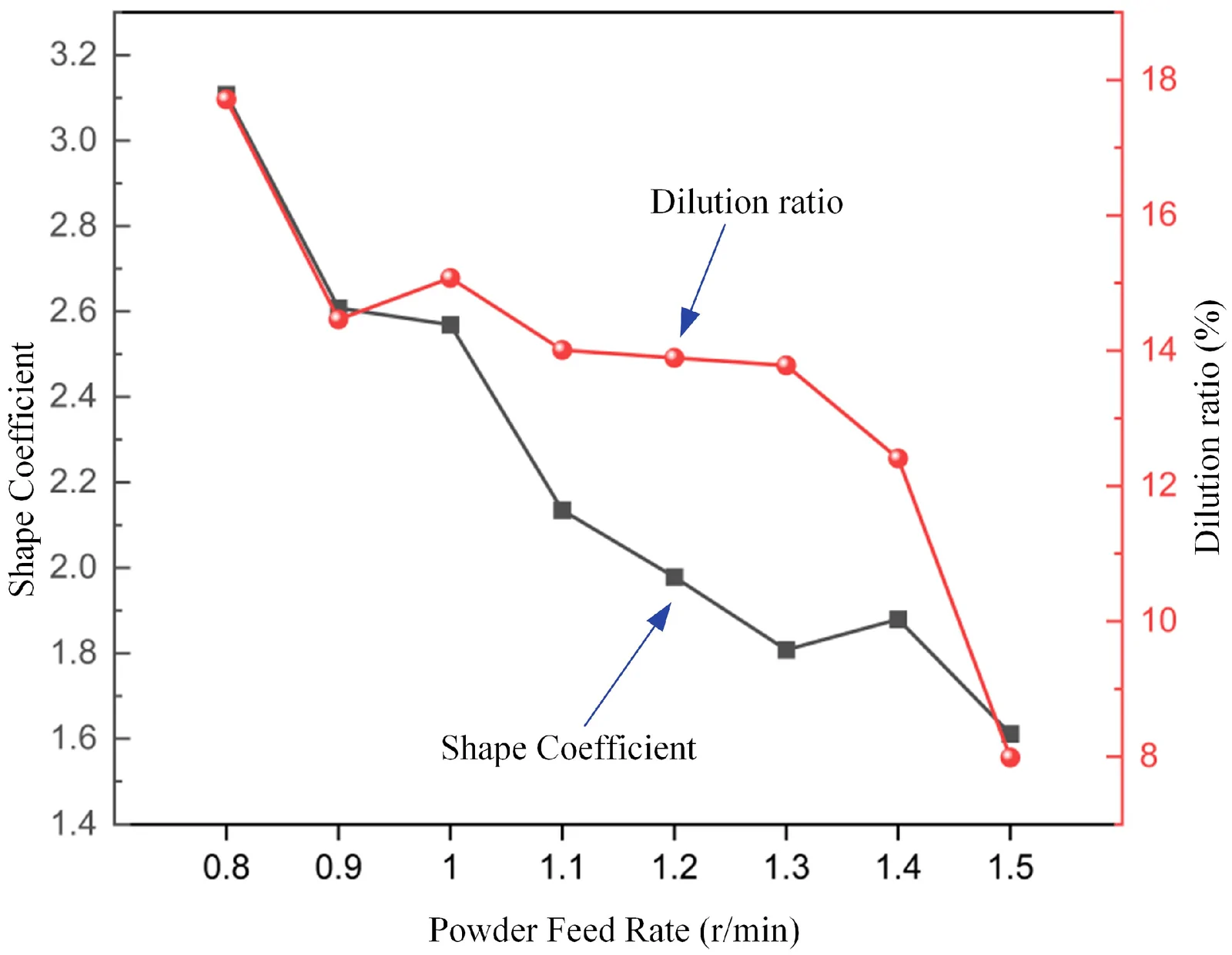
Powder feed rate versus shape factor and dilution rate.

**Figure 12:**
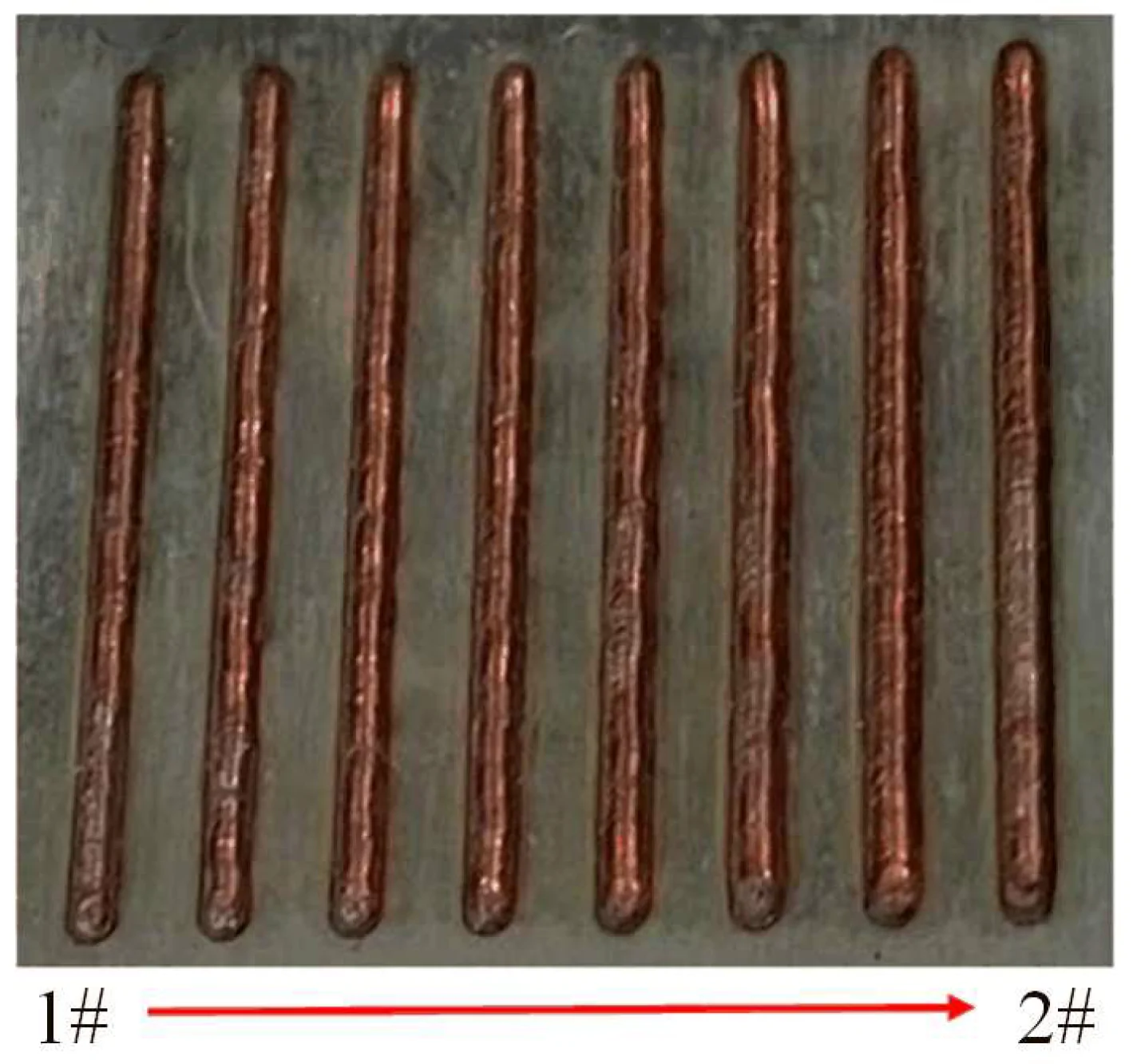
The macroscopic morphology in the single factor experiment of powder feed rate.

The relationship between the powder feed rate, the shape factor, and the dilution ratio is shown in Figure 11. It can be seen that as the powder feed rate increases from 0.8 r/min to 1.5 r/min, the shape factor decreases from 3.11 to 1.61, and the dilution ratio decreases from 17.71% to 7.98%. This is because an increase in the powder feed rate causes a significant increase in the melt height, while the melt width and melt depth remain largely unchanged. The surface morphology of the cladding layer is shown in Figure 12. When the powder feed rate is 1.1 r/min, the surface of the cladding layer is relatively smooth and continuous, with a brighter red color; no obvious defects such as powder adhesion or cracking are observed, indicating good forming quality. Taking into account that the dilution ratio should be low while ensuring good metallurgical bonding, and that the shape factor should be slightly greater than 2, orthogonal experiments were conducted using a powder feed rate of 1.1–1.3 r/min.

### 3.3. Analysis of a Single-Factor Experiment on Scanning Speed

**Figure 13:**
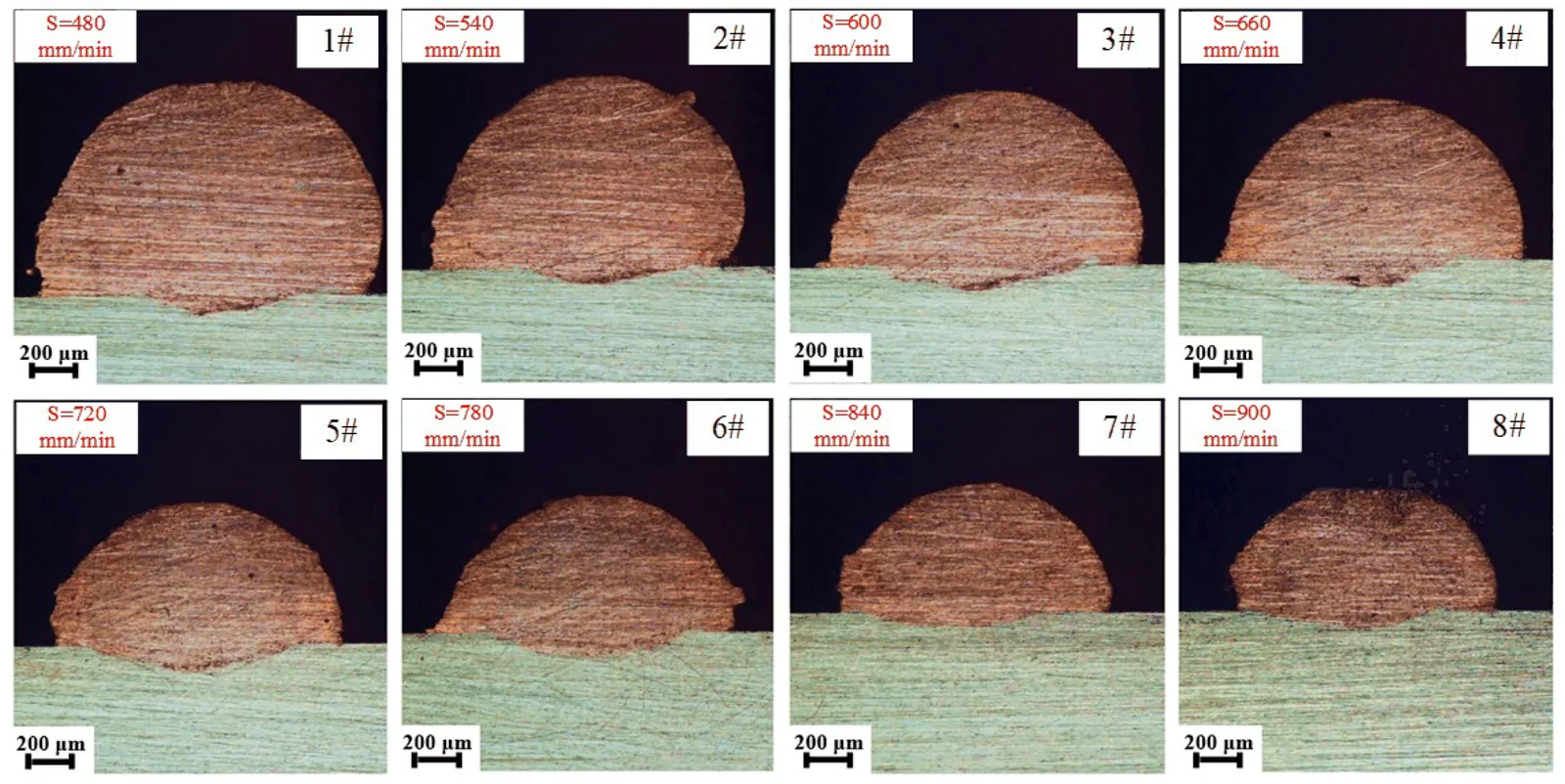
The morphology of the cladding layer in the single factor experiment of scanning speed.

Based on preliminary experiments, the experimental design and results for the single-factor scan speed experiment are shown in Table 7. Figure 13 illustrates the morphology of the cladding layer, which demonstrates that the cladding layer and the substrate form a good metallurgical bond.

**Figure 14:**
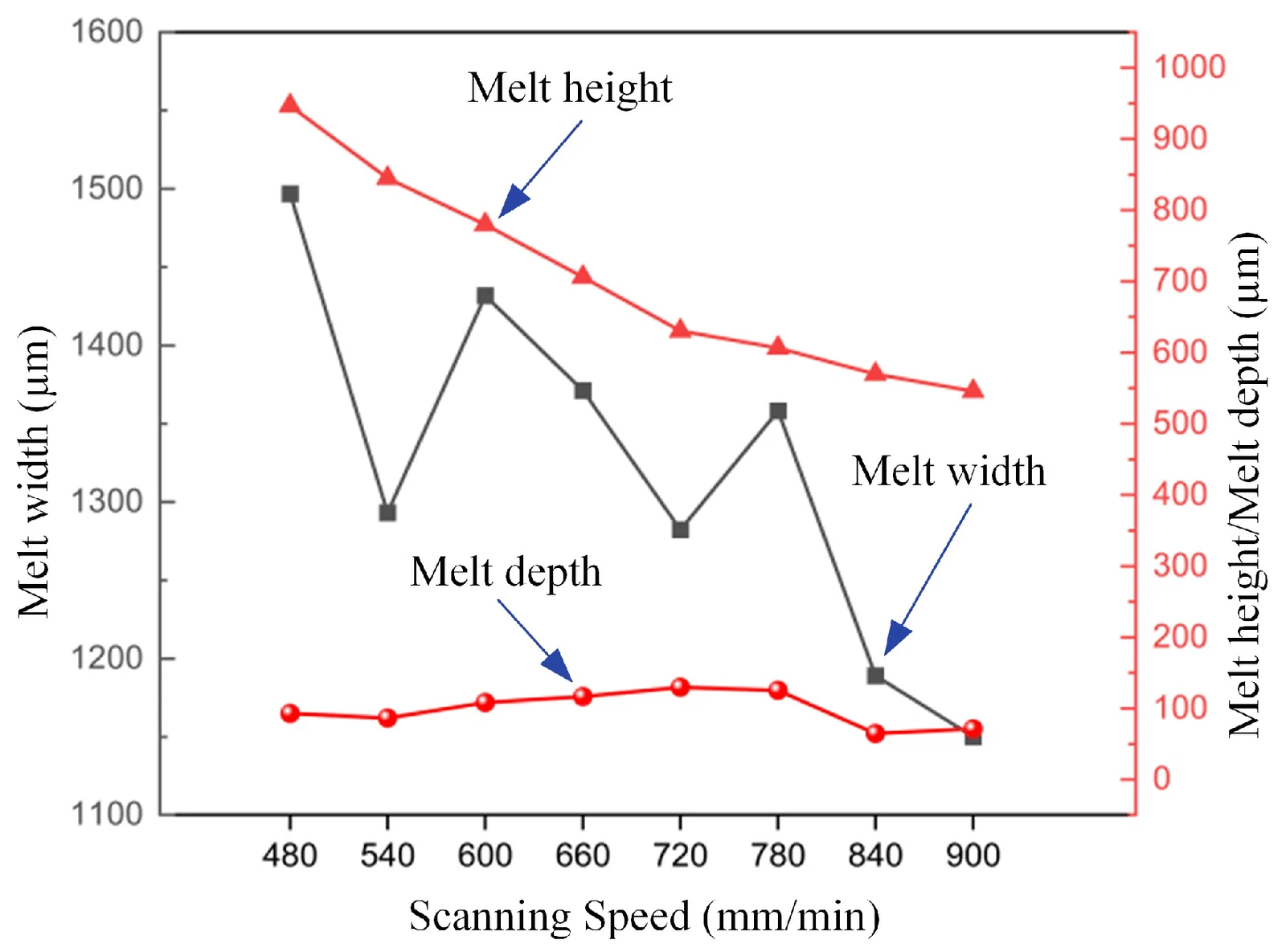
Scanning speed versus melt width, melt height, and melt depth.

The relationship between scanning speed and melt width (*W*), melt height (*H*), and melt depth (*h*) is shown in Figure 14. Under conditions of 500 W laser power and a powder feed rate of 1.3 r/min, as the scanning speed increased from 480 mm/min to 900 mm/min, the geometric parameters of the cladding layer all changed to varying degrees. Specifically, the melt width (*W*) generally showed a fluctuating downward trend, ranging from 1150.21 to 1496.81 μm. When the scanning speed was in the low range of 480–600 mm/min, the variation in melt width was significant; as the scanning speed continued to increase, although there were slight fluctuations in melt width, it generally continued to decrease. Melt height (*H*) decreased significantly as the scanning speed increased, dropping from 946.68 μm to 545.86 μm. The rate of decrease in melt height was relatively rapid in the low scanning speed range, gradually slowed as the scanning speed increased, and stabilized in the high scanning speed range. Melt depth (*h*) exhibited a fluctuating trend, first increasing and then decreasing. When the scanning speed was in the moderate range of 600–720 mm/min, the melt depth increased significantly, reaching a maximum of 129.97 μm at 720 mm/min. As the scanning speed continued to increase, the melting depth decreased significantly. As the scanning speed increased, both the powder distribution density and the laser energy density decreased simultaneously; the total amount of molten powder decreased, the heat-affected zone shrank, and the energy absorbed by the fusion zone declined—these factors were the primary causes of the aforementioned patterns.

**Figure 15:**
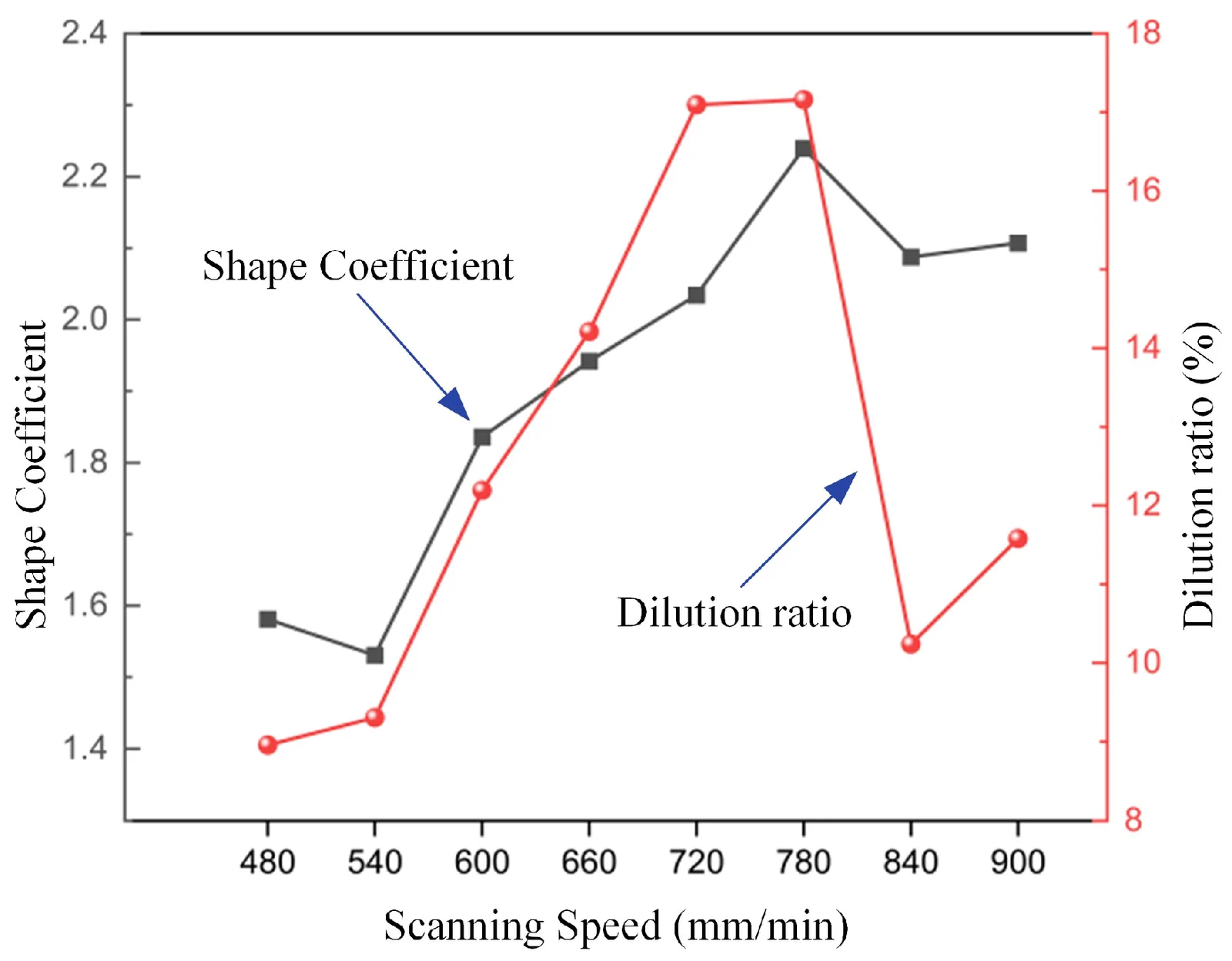
Scanning speed versus shape factor and dilution rate.

The relationship between scan speed, shape factor, and dilution ratio is shown in Figure 14. It can be seen that when the scan speed increases from 480 mm/min to 900 mm/min, the shape factor first increases from 1.58 to 2.24 and then decreases to 2.11; simultaneously, the dilution ratio increases from 8.96% to 17.16% and subsequently decreases to 11.58%. This is because, as the scanning speed increases, the melt height of the cladding layer decreases significantly, while the melt width and melt depth decrease only slightly. The scanning speed versus shape factor and dilution rate is shown in Figure 15.

**Figure 16:**
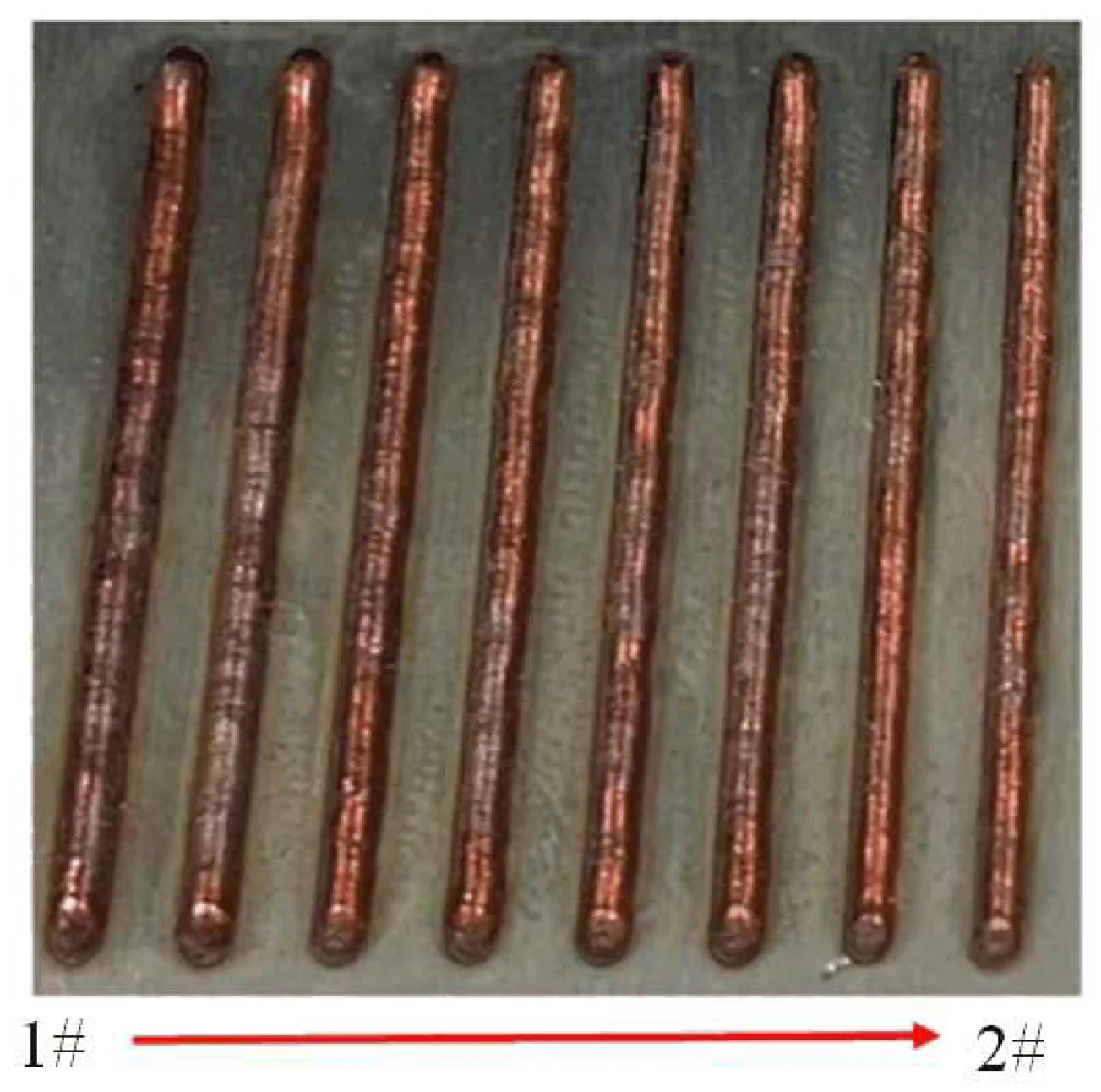
The macroscopic morphology in the single factor experiment of scanning speed.

Figure 16 presents the surface morphology of the cladding layer. When the scanning speed ranged from 600 to 840 mm/min, the surface of the cladding layer was relatively smooth and continuous, with a brighter red color. No obvious defects, such as powder adhesion or cracking, were observed, indicating good forming quality. Considering the dilution ratio, the requirement for good metallurgical bonding, and the desired shape factor of approximately 2, the scanning speed for the orthogonal experiments was set in the range of 600–800 mm/min.

## 4. Experimental Study of Laser Cladding Using an Orthogonal Design

By conducting a one-factor experiment, the range of process parameters was narrowed. To further optimize the laser cladding process parameters, a three-factor, five-level orthogonal experiment was conducted using an L25 (5^3^) orthogonal array. The experimental factors and levels are shown in Table 8.

### 4.1. Results of the Orthogonal Experiment

**Figure 17:**
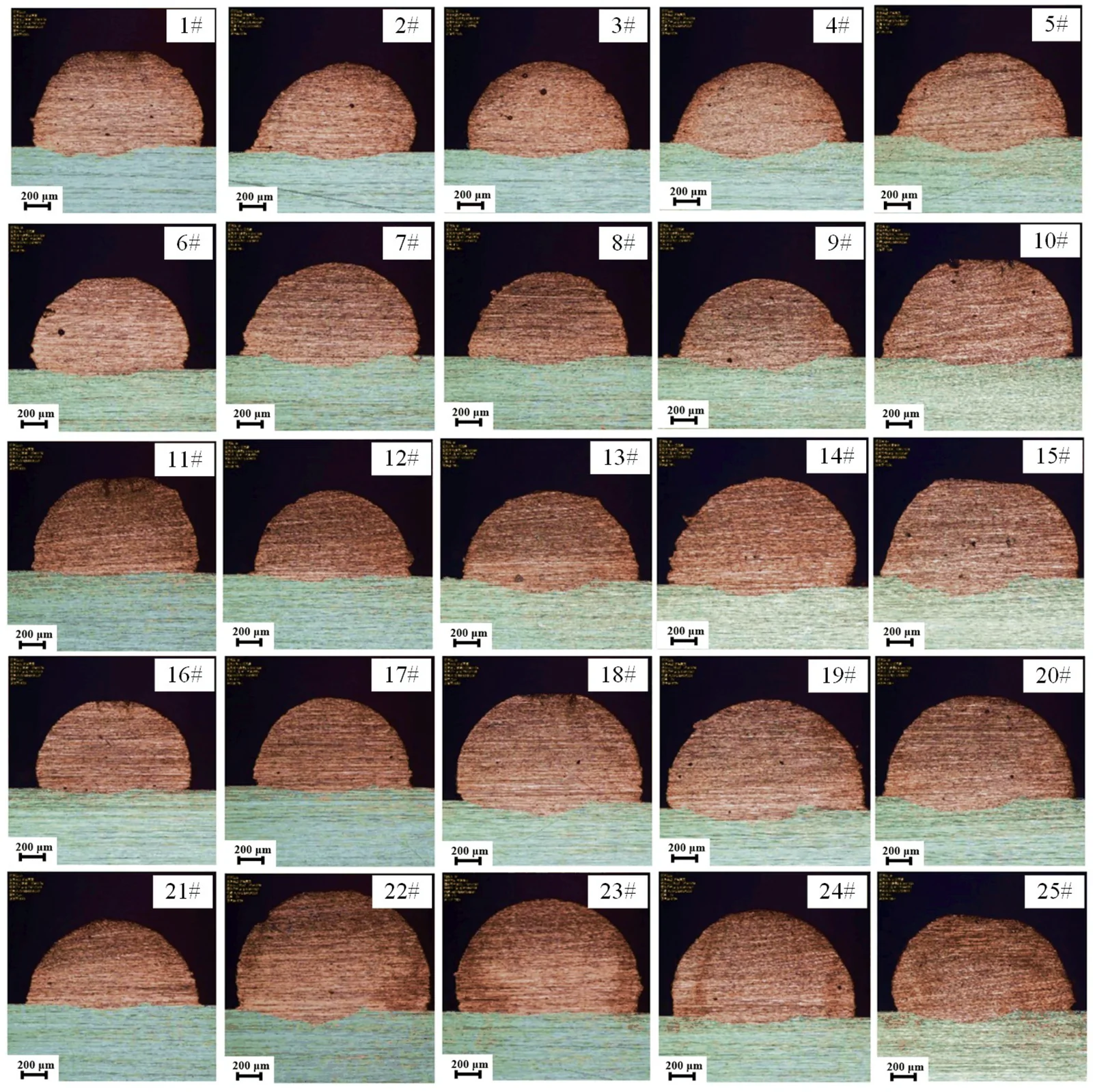
Cross-sectional microscopic morphology.

**Figure 18:**
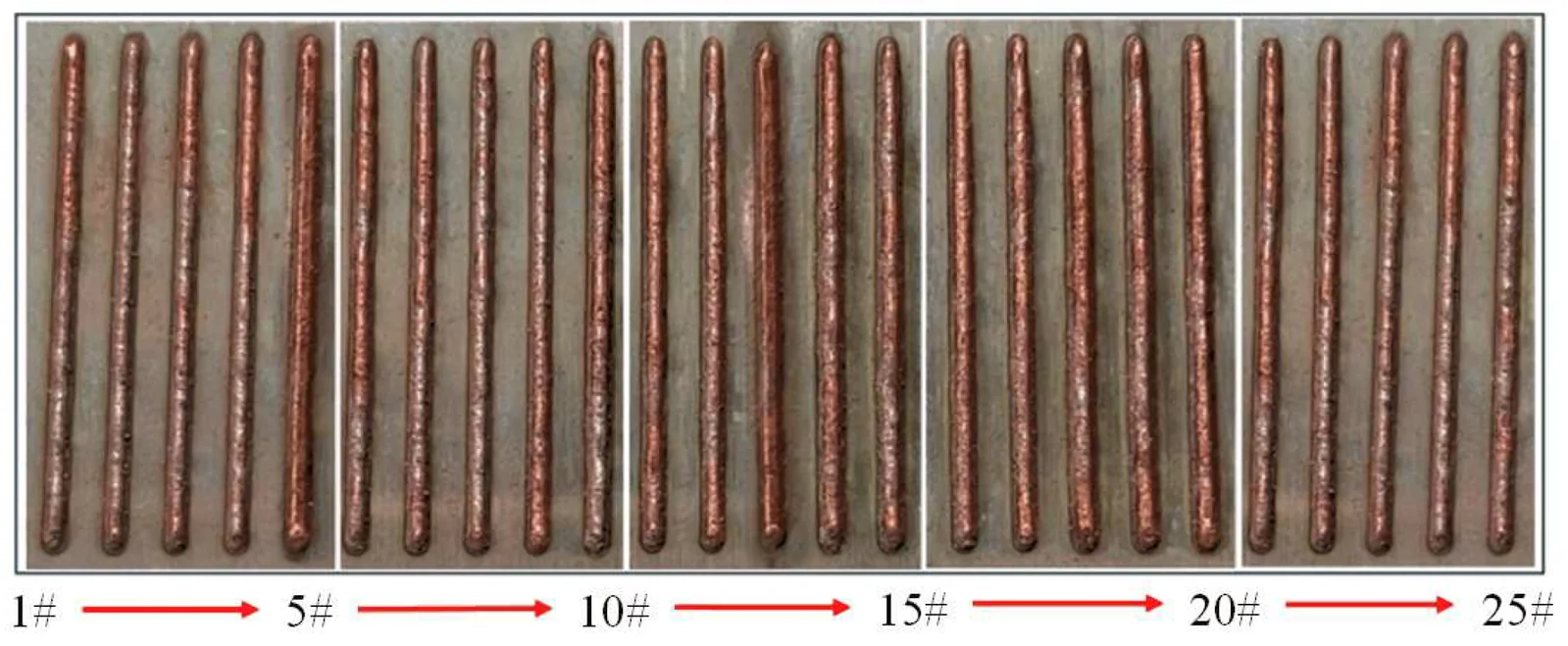
Macroscopic morphology.

Orthogonal experimental scheme and results is shown in Table 9. Figure 17 denotes the cross-sectional microscopic morphology. Macroscopic morphology is also an important indicator for evaluating cladding quality. As shown in Figure 18, Sample 5 has a smoother surface and a brighter red color, with no severe powder adhesion or cracking observed. In summary, the optimal sample is Sample 5, with the following parameter combination: laser power of 500 W, powder feed rate of 1.1 r/min, and scan speed of 800 mm/min, denoted as *P*5*F*1*S*5. The next best sample is Sample 13, with the parameter combination of laser power of 450 W, powder feed rate of 1.2 r/min, and scan speed of 750 mm/min, denoted as *P*3*F*3*S*5.

### 4.2. Analysis of Range in Orthogonal Experiments

Range analysis can be used to qualitatively determine the extent to which process parameters affect the shape coefficient and dilution ratio by comparing the ranges of different process parameters. If the range of a particular parameter is large, it indicates that when that parameter varies within a certain range, the values of the shape coefficient or dilution ratio change significantly, meaning it has a significant impact on the experimental indicators. If the range of a particular parameter is small, it indicates that its impact on the experimental indicators is minimal; therefore, the magnitude of a process parameter’s influence on the shape coefficient and dilution ratio can be determined based on the value of its range.

Table 10 presents the results of the range analysis for the shape coefficient. It can be seen that, assuming no interactions are considered, the factors influencing the shape coefficient, in descending order of significance, are scan speed *S*, powder feed rate *F*, and laser power *P*. The optimal combination derived from this is *P*5*F*1*S*5.

Table 11 presents the results of a range analysis of the dilution rate. It can be seen that, assuming no interactions, the factors affecting the dilution rate, in descending order of influence, are: laser power *P*, powder feed rate *F*, and scan speed *S*. The optimal combination derived from this analysis is *P*5*F*1*S*5.

**Figure 19:**
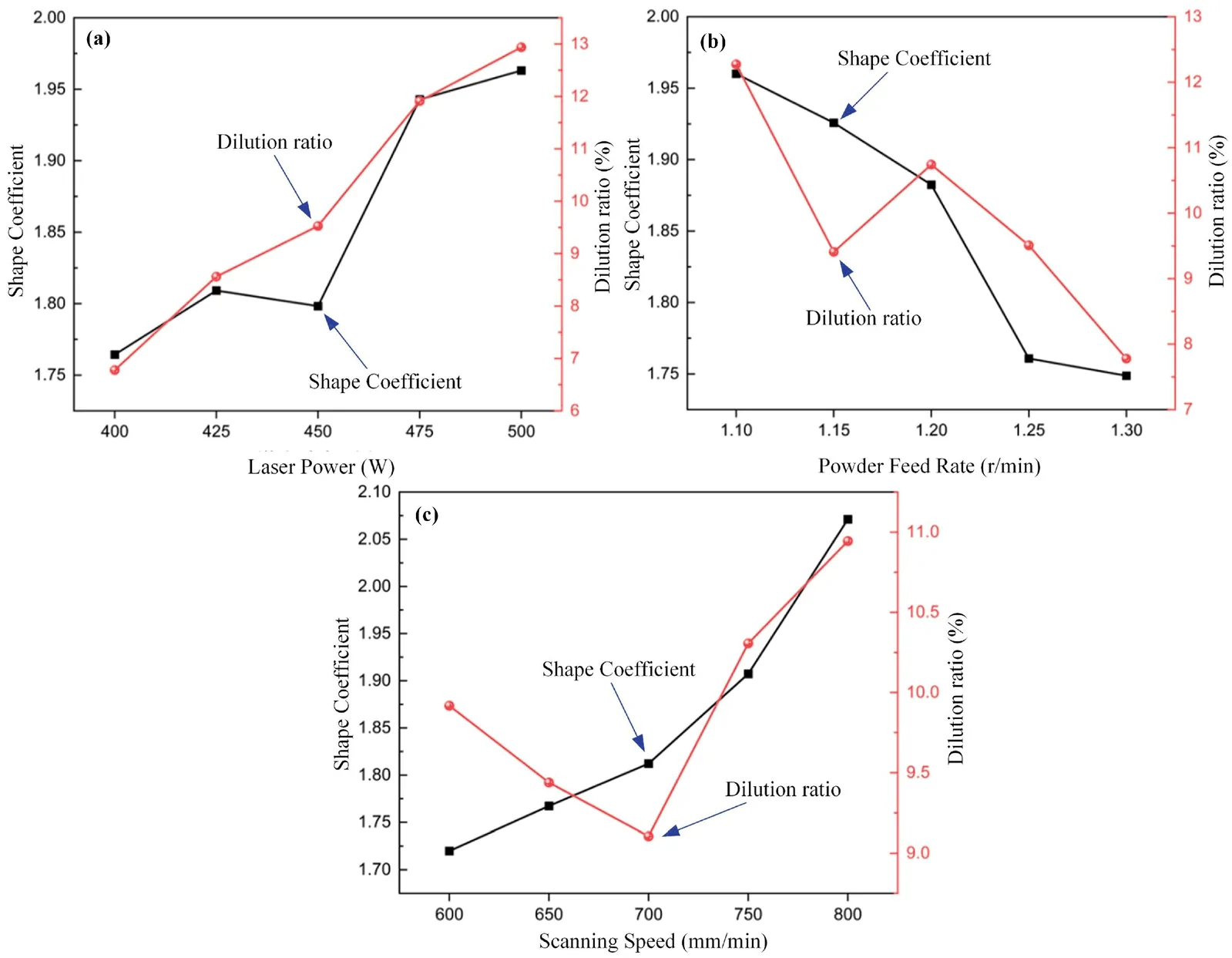
Process parameters with mean values of shape factor and dilution rate (**a**) laser power, (**b**) powder feeding rate, (**c**) scanning speed.

Figure 19 shows the relationship between process parameters and the mean values of the shape factor and dilution ratio. Based on the results of the range analysis, it can be seen that, for the dilution ratio, the order of influence of the factors is *P* > *F* > *S*, while for the shape factor, the order is *S* > *F* > *P*. It can be observed that as laser power *P* increases, the trends in the shape factor and dilution ratio are not pronounced. Specifically, the dilution ratio shows a slight upward trend, while the shape factor remains largely unchanged. As powder feed rate *F* increases, both the shape factor and dilution ratio gradually decrease, indicating that this factor has a relatively significant impact on both metrics. As scanning speed *S* increases, both the shape factor and dilution ratio gradually increase, with the upward trend in the shape factor being more pronounced. Overall, the trends in the various process parameters observed in the orthogonal experiment are generally consistent with the results of the range analysis. This indicates that laser power primarily affects the dilution ratio, scan speed has a significant impact on the shape factor, and powder feed rate exerts a moderate influence on both parameters.

### 4.3. Analysis of Variance for Orthogonal Experiments

Range analysis provides the relative ranking of process parameters according to their effects on the selected cladding quality responses; however, it does not distinguish the contribution of individual factors from random experimental variations. To further evaluate the statistical significance of each parameter and reduce the influence of experimental errors, analysis of variance (ANOVA) was subsequently performed. The significance of the factors was assessed based on the *p*-value, with the corresponding criteria summarized in Table 12.

According to the ANOVA results presented in Table 13, scanning speed contributes significantly to the variation in the shape coefficient, while the effects of laser power and powder feed rate are statistically negligible. The significance ranking obtained from ANOVA is in accordance with that derived from the range analysis. In Table 13, Adj SS represents adjusted sum of squares, Adj MS represents adjusted mean square. Adj SS and Adj MS are the core statistical measures used to evaluate the significance of the impact of various factors.

**Figure 20:**
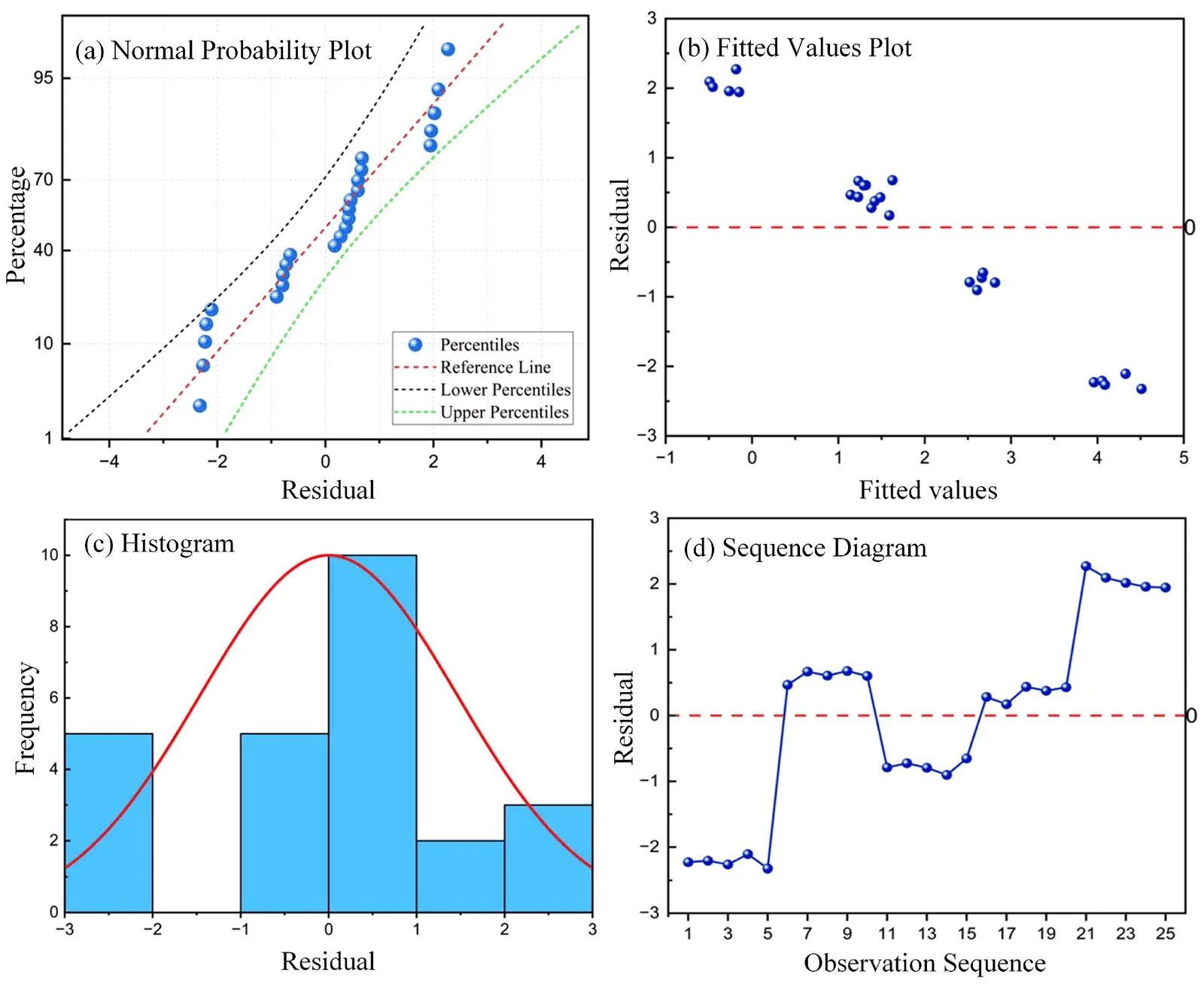
Residual plots of shape coefficients.

Figure 20 shows the residual plot for the shape coefficient. As can be seen from the normal probability plot in Figure 20a, the residual points are distributed fairly uniformly around the line *y* = *x*, indicating that the residuals follow a normal distribution and that the model fits the data well. In the fitted values plot (Figure 20b), the residual points are randomly distributed on both sides of the zero line without any obvious trend, indicating that the residual variance is essentially constant and that the model does not exhibit any significant systematic bias. The histogram (Figure 20c) shows that the residual distribution approximates a bell curve, consistent with the characteristics of a normal distribution; although there are a few outliers, their overall impact is negligible. The sequence plot in Figure 20d indicates that the residuals fluctuate with the order of observation but show no obvious pattern, suggesting that the residuals are independent of one another and exhibit no autocorrelation. Based on the above analysis, the regression model for the shape coefficient can be considered valid. Its residuals satisfy the basic assumptions of normality, independence, and homoscedasticity. Therefore, the analysis results are highly reliable and can be used for the optimization of process parameters.

Table 14 shows the shape factor values for different combinations of scanning speed and powder feed rate. Based on the previous analysis, the morphology quality of the cladding layer is optimal when *δ* > 2 and close to 2. Therefore, *δ* > 2 and close to 2 is used as the criterion for selecting process parameters. As shown in the table, there are many combinations that satisfy *δ* > 2 when the scanning speed *S* = 800 mm/min; thus, the scanning speed is set to 800 mm/min. Further analysis of the powder feed rate reveals that when *F* = 1.1 r/min and *F* = 1.2 r/min, there are two combinations in each case that satisfy *δ* > 2 and are close to 2. Considering that the powder feed rate of *F* = 1.1 r/min is lower and offers better economic efficiency, and in conjunction with the aforementioned analysis results, the powder feed rate is ultimately set to 1.1 r/min.

Considering the effects of individual processing variables on the shape factor, the preferred parameter setting was obtained at a powder feed rate of 1.1 r/min and a scanning speed of 800 mm/min. The selected condition corresponds well with the trends revealed by the orthogonal test results.

As shown by the results of the analysis of variance for the dilution ratio in Table 15, the *p*-values for laser power (*P*), scanning speed (*S*), and powder feed rate (*F)* are all greater than 0.05, indicating that the effects of these factors on the dilution ratio are not statistically significant. However, in terms of contribution rates (%), the degree of influence of each factor still varies, the factor significance order is scanning speed (*S*), powder feed rate (*F*), and laser power (*P*), with *S* > *F* > *P*. This is consistent with the results of the range analysis.

**Figure 21:**
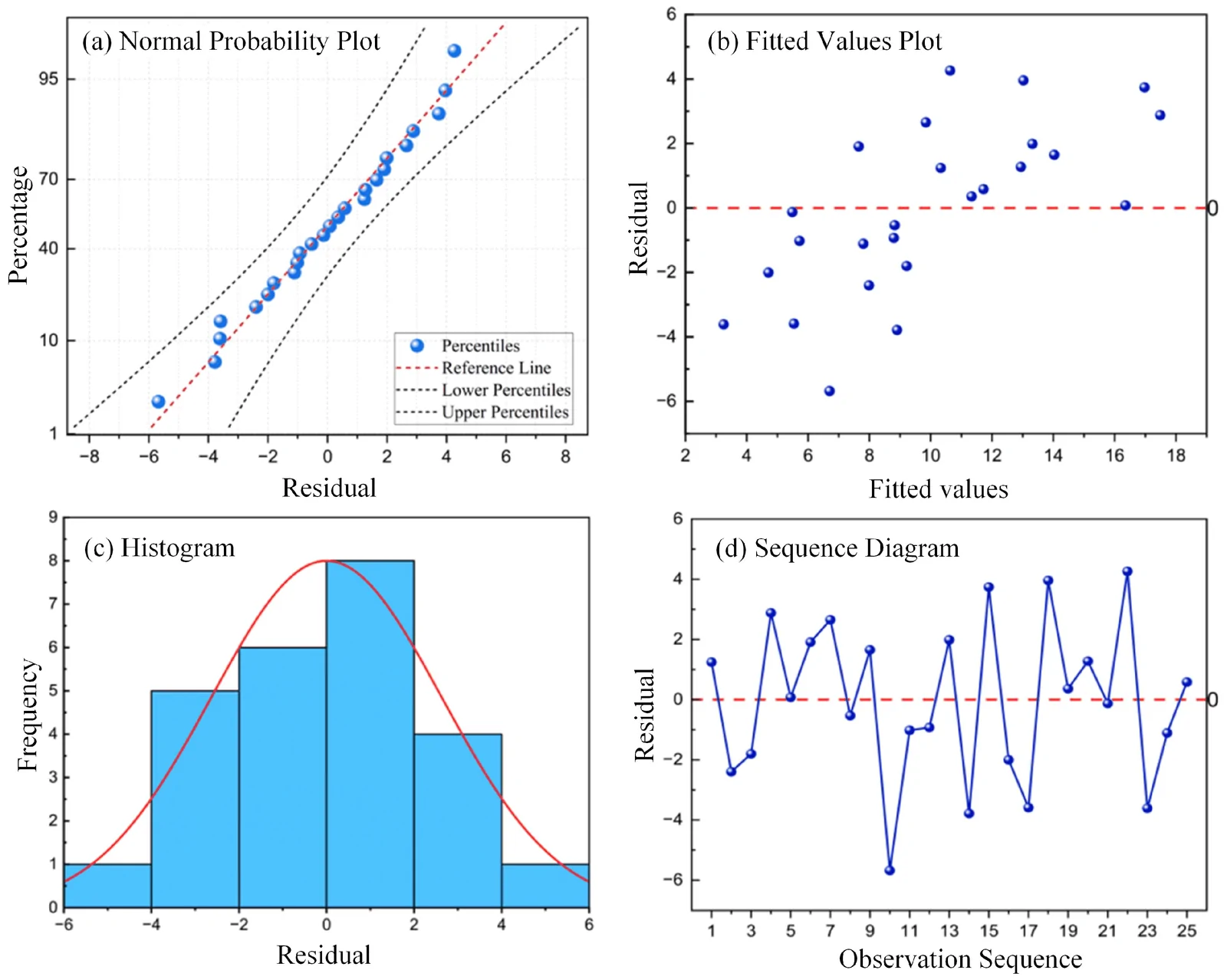
Residual plot of dilution rate.

Figure 21 shows the residual plot for the dilution ratio. As can be seen from the normal probability plot in Figure 21a, the residuals are distributed fairly uniformly around the line *y* = *x*, indicating that the residuals follow a normal distribution and that the model fits the data well. In the fitted values plot (Figure 21b), the residual points are randomly distributed on both sides of the zero line without any obvious trend, indicating that the residual variance is essentially constant and that the model does not exhibit any significant systematic bias. The histogram (Figure 21c) shows that the residual distribution approximates a bell curve, consistent with the characteristics of a normal distribution; although there are a few outliers, their overall impact is negligible. The sequence plot in Figure 21d indicates that the residuals fluctuate with the order of observation but show no obvious pattern, suggesting that the residuals are independent of one another and exhibit no autocorrelation. Based on the above analysis, the regression model for the dilution rate can be considered valid, as its residuals satisfy the basic assumptions of normality, independence, and homoscedasticity. Therefore, the analysis results are highly reliable and can be used for the optimization of process parameters.

Since the analysis of variance for the shape factor has already identified some optimal combinations of scanning speed and powder feed rate, this section focuses on analyzing the laser power and powder feed rate. Table 16 shows the dilution ratios for different combinations of these two parameters. As indicated by the previous analysis, the cladding layer morphology is relatively ideal when 13 < *η* < 17; therefore, 13 < *η* < 17 is adopted as the selection criterion for process parameter combinations. Based on the aforementioned analysis results, the optimal powder feed rate was determined to be *F* = 1.1 r/min. Under these conditions, the corresponding dilution ratio *η* = 16.35%, which meets the requirements, and the corresponding laser power is *P* = 500 W. The insensitivity of the dilution ratio to variations in laser power and scanning speed may be attributed to the exceptionally high thermal conductivity of the CuCrZr copper alloy. The high thermal conductivity facilitates rapid lateral heat dissipation from the melt pool, mitigating the impact of fluctuations in linear energy density on melt depth, and thereby endowing this material system with robust dilution-ratio stability over a wide parameter window.

The combined evaluation of shape factor and dilution ratio indicates that *P*5*F*1*S*5 is the most favorable processing condition, with a laser power of 500 W, powder feed rate of 1.1 r/min, and scanning speed of 800 mm/min. The obtained optimal condition is consistent with the tendency predicted by the orthogonal test analysis.

### 4.4. The Morphologies of the Wear Tracks for Both the Coating and the Substrate

The friction and wear tests were conducted to evaluate the wear resistance of the laser cladding coatings and the substrate. The experiment was conducted at room temperature. A multifunctional tribometer (MFT-4000, Lanzhou Huahui Instrument Technology Co., Ltd., Lanzhou City, Gansu Province, China) was employed for the wear experiments. An Al_2_O_3_ ceramic ball was used as the counter body. The tests were performed under a normal load of 10 N, a reciprocating sliding speed of 220 mm/min, and a sliding length of 4 mm. The wear tracks were subsequently analyzed to investigate the wear behavior and mechanisms of the coatings and substrate.

**Figure 22:**
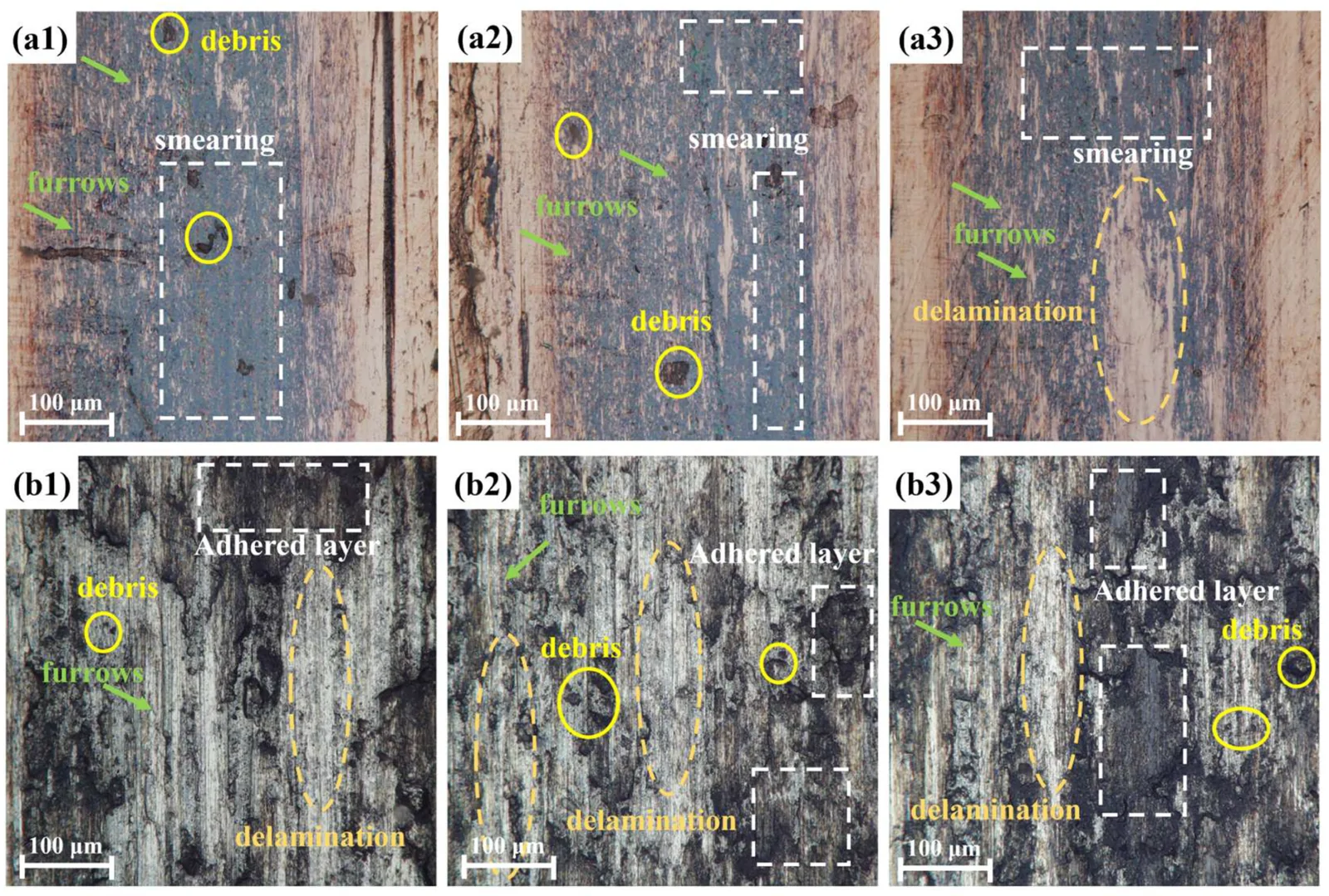
Wear track morphologies: (**a1**–**a3**) coating; (**b1**–**b3**) substrate.

Figure 22 presents the morphologies of the wear tracks for both the coating and the substrate. The wear surface of the CuCrZr coating is characterized by extensive smearing, accompanied by limited furrows and delamination features. This indicates that the wear mechanism is dominated by plastic deformation. The intrinsic ductility of the Cu-based coating facilitates material flow under shear stress, leading to the formation of a smeared surface layer. This layer acts as a protective tribolayer, which accommodates deformation, reduces stress concentration, and suppresses crack initiation and propagation.

The wear surface of the 316L substrate exhibits pronounced adhesive wear, along with more severe delamination and deeper furrow features. The repeated adhesion and rupture at the sliding interface lead to the formation of micro-junctions between contacting asperities. Under shear stress, these junctions are fractured, resulting in material transfer and removal, thereby giving rise to adhesive wear. Meanwhile, subsurface stress accumulation promotes crack initiation beneath the surface, leading to delamination. Some detached wear debris are entrapped within the contact interface, forming a three-body abrasion system that further aggravates surface damage and contributes to the development of furrows.

In summary, the superior wear resistance of the Cu-based coating can be attributed to its high ductility and the formation of a smeared tribolayer during sliding. The coating effectively accommodates plastic deformation and reduces interfacial shear stress, thereby minimizing direct metal-to-metal contact and wear damage. Therefore, the laser-cladded Cu-based coating significantly improves the surface wear resistance of the 316L substrate.

### 4.5. Analysis of Process Parameter Interactions and Discussion of Response Patterns

The aforementioned range and variance analysis has determined the order of importance of each process parameter’s influence on the shape coefficient and dilution ratio, as well as the optimal parameter combinations. In terms of the response pattern of the shape coefficient, scanning speed has the most significant effect (40.85% contribution), followed by powder feed rate, while laser power has the least influence. However, the variation in the shape coefficient under different parameter combinations is not a simple superposition of the effects of individual factors. Taking the interaction between scan speed and powder feed rate as an example, when the scan speed is at a lower level (600–650 mm/min), increasing the powder feed rate from 1.1 r/min to 1.3 r/min causes the shape coefficient to decrease from 1.73–1.84 to 1.56–1.61, a reduction of approximately 10–15%. However, when the scanning speed is at a higher level (750–800 mm/min), within the same range of increase in powder feed rate, the shape coefficient decreases from 2.22–2.19 to 1.80–2.09, with a significantly narrower reduction. This indicates that under high scanning speed conditions, the laser energy density received per unit length of the substrate surface decreases. The reduced amount of powder melted, and the slowed growth rate of the melt height thereby weaken the regulatory effect of the powder feed rate on the shape factor. In other words, there is a clear antagonistic interaction between the scanning speed and the powder feed rate. The former indirectly modulates the latter’s influence on the geometric morphology of the cladding layer by altering the laser energy input density. This finding implies that, in the practice of process parameter optimization, the impact of the powder feed rate on forming quality should not be discussed in isolation from the scanning speed conditions.

The response pattern of the dilution rate exhibits distinct interactive characteristics. Range analysis indicates that laser power has the most significant effect on the dilution rate, followed by powder feed rate, while scan speed has a relatively weaker influence. Further examination of the combined effect of laser power and powder feed rate reveals that when the laser power is 400 W, the dilution rate decreases as the powder feed rate increases (from 10.33% to 5.49%). This is because, under low-power conditions, energy input is limited; an increase in powder feed rate causes more laser energy to be used for melting the powder. At this point, the effective energy transferred to the substrate decreases, and the melt depth correspondingly decreases. However, when the laser power is increased to 500 W, the dilution ratio exhibits a non-monotonic fluctuation pattern—first decreasing, then increasing, and then decreasing again—in response to changes in the powder feed rate. This indicates that, under conditions of sufficient energy supply, the effect of powder feed rate on the dilution ratio is subject to multiple constraints arising from changes in the melt pool flow state and powder capture efficiency; the relationship between the two is not a simple linear negative correlation. In the analysis of variance, the *p*-values for all three process parameters were greater than 0.05, indicating that the dilution ratio is relatively insensitive to these parameters. This may be due to the high thermal conductivity of the CuCrZr copper alloy, which results in a more uniform temperature distribution within the melt pool. Changes in heat input caused by fluctuations in a single parameter are partially buffered by the material’s own thermal diffusion behavior, thereby keeping the dilution ratio relatively stable within a wide process window. This characteristic offers certain advantages for engineering applications. The dilution ratio’s high tolerance to parameter fluctuations helps reduce the difficulty of process control during mass production.

From an optimization perspective, the optimal parameter combination *P*5*F*1*S*5 identified in this study achieves a good balance between the shape factor and the dilution ratio. It is worth noting that Sample 15 (*P*5*F*1*S*5) achieved a dilution ratio of 16.98%, which is close to that of the optimal sample. However, its shape factor was only 2.02, and the contour of its cladding layer was closer to an ideal arc shape. Taking both the dilution ratio and the shape factor into comprehensive consideration, the optimal scheme P5F1S5 prioritizes the improvement in cladding efficiency achieved through high scanning speed, whereas Sample 15 achieves a superior contour shape through the combination of moderate scanning speed and a slightly higher powder feed rate. This analysis indicates that in practical engineering applications, a trade-off should be made between cladding efficiency (prioritizing *P*5*F*1*S*5) and forming accuracy (considering *P*5*F*3*S*2) based on specific requirements, rather than mechanically applying a single optimal combination. Furthermore, the overlap ratio and *Z*-axis lift corresponding to Sample 15 can be recalculated using a mathematical model to establish parameter-matching strategies tailored to different quality objectives.

## 5. Determining the Overlap Ratio and Increase Amount

Single-pass cladding is limited by the laser spot diameter, resulting in a limited cladding width. Larger cladding areas can be achieved through multi-pass overlapping and multi-layer overlapping. In this section, the theoretical values of the overlap ratio and packing density will first be determined through theoretical derivations, and then the optimal value of the overlap ratio will be verified experimentally.

### 5.1. Determining the Lap Ratio

The overlap ratio is the percentage of the total weld width that is remelted in adjacent weld passes during multi-pass welding. It is also commonly expressed as the center-to-center distance between adjacent weld passes. The formula is as follows:

(4) $$\lambda =\frac{W-d}{W}$$

In Equation (4), *d* represents the center-to-center distance between adjacent melt paths, and *W* represents the width of a single melt path.

**Figure 23:**
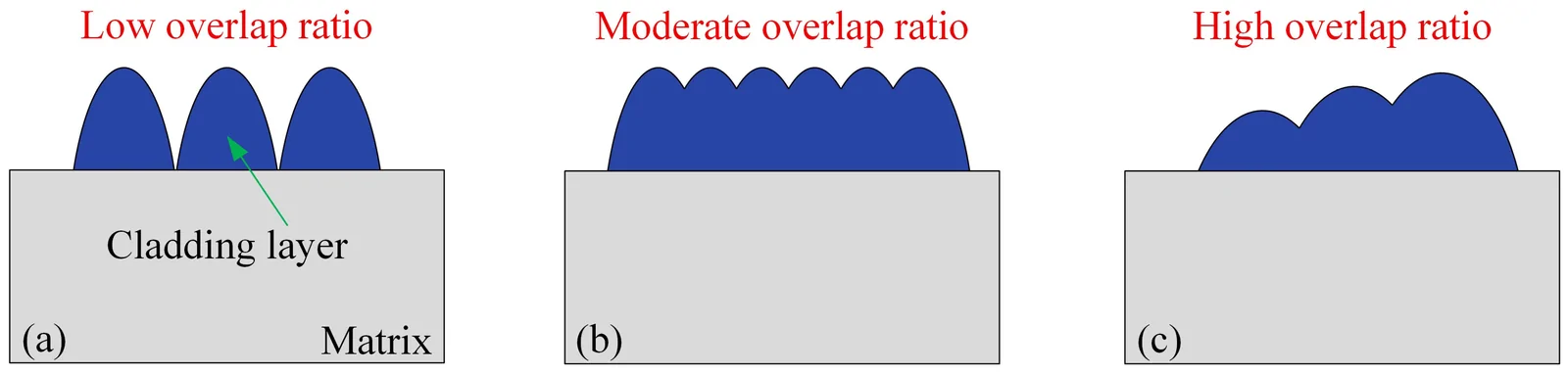
Several typical lap shapes: (**a**) low overlap ratio, (**b**) Moderate overlap ratio, (**c**) High overlap ratio.

**Figure 24:**
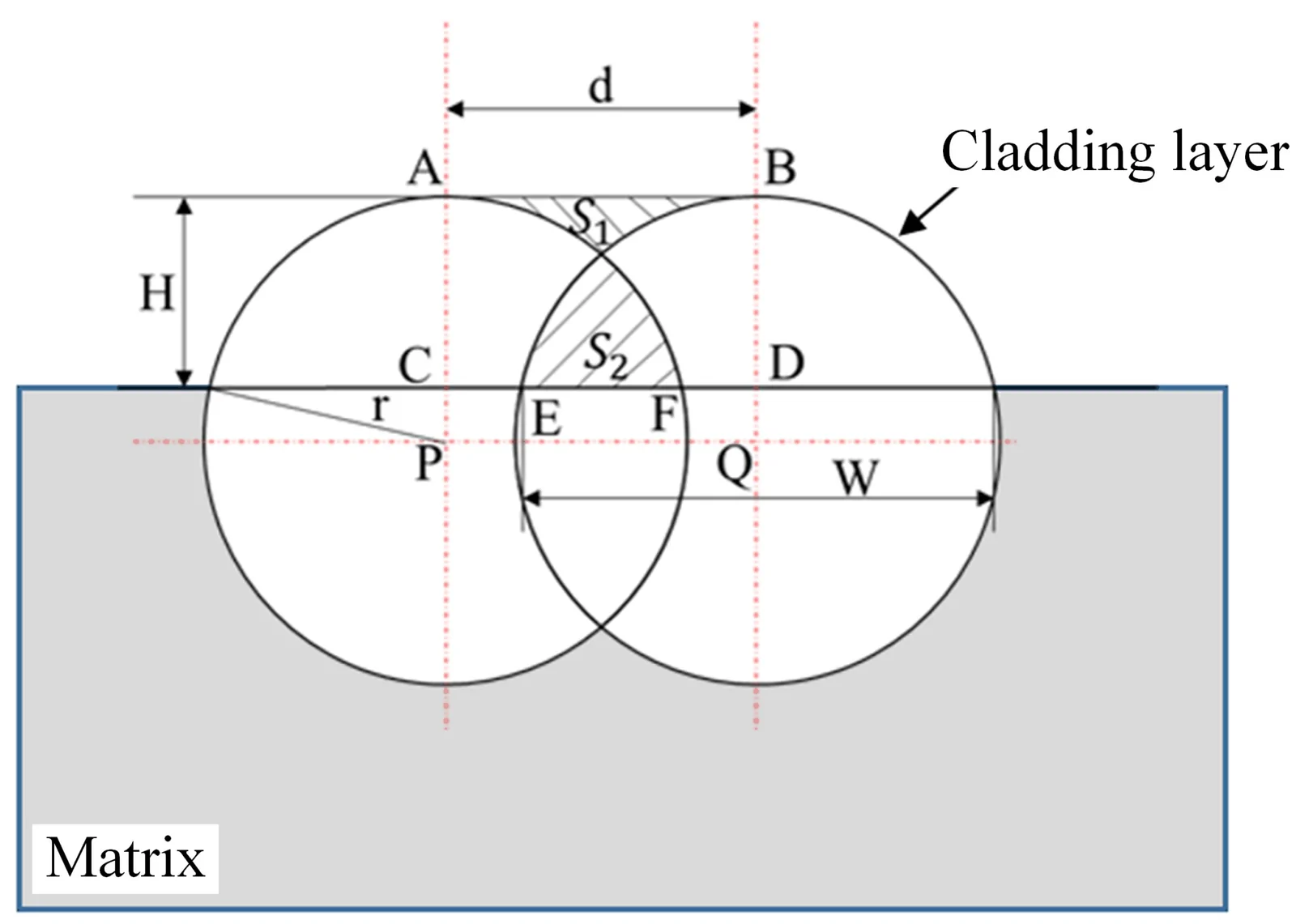
Theoretical overlap ratio model.

Figure 23a shows that when the overlap ratio is too small, the surface topography becomes noticeably uneven, causing the cladding layers to collapse and preventing effective bonding between the two passes. Figure 23b shows a moderate overlap ratio, where the surface of the cladding layer is generally flat, resulting in good cladding performance and facilitating subsequent cladding. Figure 23c illustrates that if the overlap ratio is too high, the overlapping area between the two passes becomes excessive, causing the cladding layer to build up excessively and fail to meet processing requirements. This should be avoided as much as possible. Figure 24 shows a theoretical overlap ratio model. The theoretical overlap ratio model was established based on two assumptions: the cross-section of a single cladding track is approximated as a circular segment, and adjacent tracks possess the same processing conditions and surface geometry [26,27].

The relationship between the radius (*r*) of the circle inscribed around the melt cross-section and the melt width *W* and melt height *H* is as follows:

(5) $$r=\frac{4H^{2}+W^{2}}{8H}$$

Ideally, after adjacent weld beads are joined, the molten metal in the remelted region *S*_2_ should exactly fill the gap between the adjacent weld beads—that is, region *S*_1_. Assume that the area of region *S*_2_ is equal to that of region *S*_1_:

(6) $$S_{\mathrm{ABCD}}=S_{\mathrm{ACF}}+S_{\mathrm{BDE}}$$

The area of region ACF can be calculated using the formula for the area of a sector:

(7) $$S_{\mathrm{ACF}}=S_{\mathrm{BDE}}=\frac{1}{2}{\left(\frac{4H^{2}+W^{2}}{8H}\right)}^{2}\arcsin(\frac{4WH}{4H^{2}+W^{2}})-\frac{W(W^{2}-4H^{2})}{32}$$

Substituting Equation (7) into Equation (4) yields the relationship between the theoretical overlap ratio *λ* and the weld width *W* and weld height *H*:

(8) $$\lambda =1-\frac{{\left(4+W^{2}/H^{2}\right)}^{2}}{64W/H}\arcsin(\frac{4W/H}{4+W^{2}/H^{2}})+\frac{W^{2}/H^{2}-4}{16}$$

Furthermore, since *W*/*H* = *δ*, conclude that:

(9) $$\lambda =1-\frac{{\left(4+\delta^{2}\right)}^{2}}{64\delta }\arcsin(\frac{4\delta }{4+\delta^{2}})+\frac{\delta^{2}-4}{16}$$

According to the orthogonal test results, the optimized parameters were determined as 500 W laser power, 1.1 r/min powder feed rate, and 800 mm/min scanning speed. The melt width is 1.341 mm, the melt height is 0.613 mm, and the shape factor *δ* is 2.186. Substituting these values into the above Equation (9), the theoretical overlap ratio *λ* is found to be 23%, and the center-to-center distance d between adjacent melt tracks is 1.03 mm. To investigate the overlap ratio, five sets of parameters were selected within the upper and lower ranges of this value: 0.7 mm, 0.8 mm, 0.9 mm, 1.03 mm, and 1.06 mm. The experimental design is shown in Table 17.

**Figure 25:**
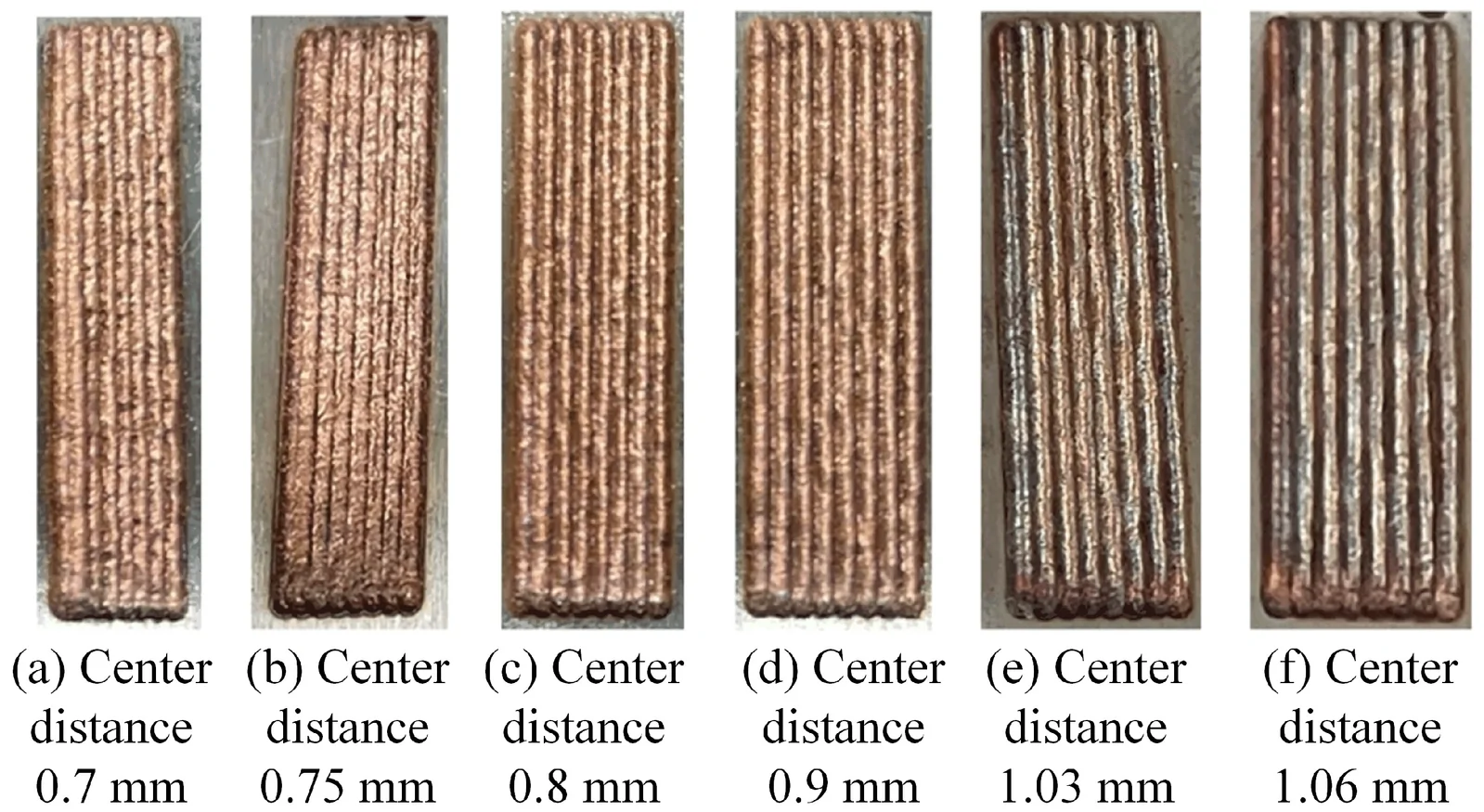
Macroscopic morphology of overlap surfaces at different center distances.

**Figure 26:**
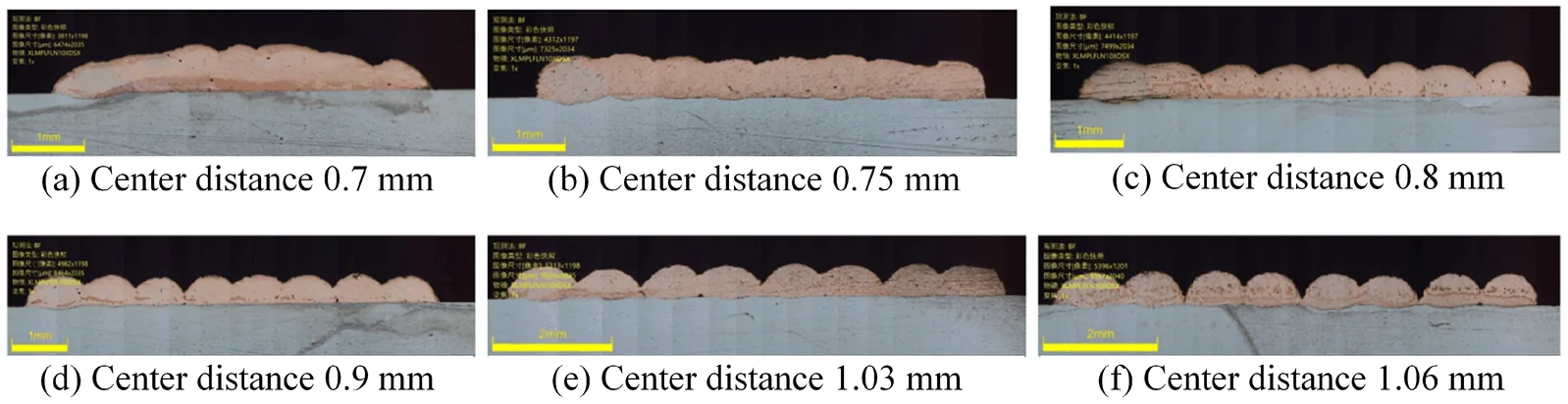
Confocal morphology at different center distances.

The macroscopic morphology of the overlap at different center distances is shown in Figure 25 and Figure 26 below. As the center distance increases, the width of the overlap deposit gradually increases, while the surface flatness improves. When the center distance is 0.75 mm—a value corresponding to an overlap ratio *λ* of 44%—the surface of the cladding layer is relatively flat and free of obvious depressions. This indicates that the selection of this center distance is reasonable.

The theoretical calculation yielded an overlap ratio of 23%, whereas the experimental measurement resulted in 44%. The primary cause of this discrepancy lies in the theoretical model’s idealization of the molten pool morphology (e.g., assuming a regular geometric shape). Additionally, measurement errors and instability in powder transport also exerted a certain influence on the results.

### 5.2. Derivation of the Ideal Increase

**Figure 27:**
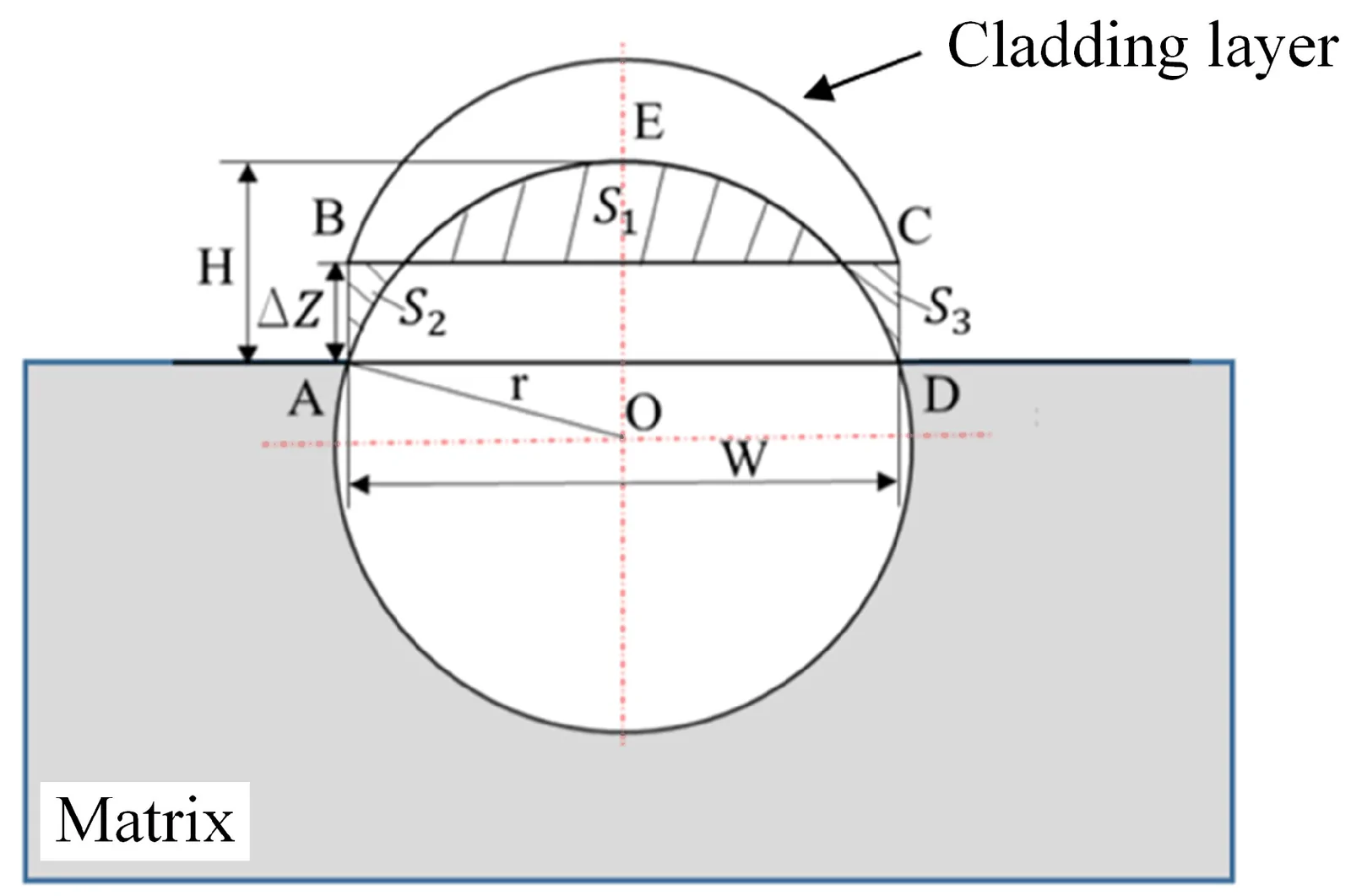
Model of lifting volume.

The lift amount is a key factor affecting the quality of laser multi-layer cladding. During actual cladding, the previous cladding layer serves as the substrate for the current layer. Under the combined effects of surface tension and gravity, the molten metal in the remelted area flows along both sides of the previous cladding layers’ trajectory, causing the height to fall short of the set value. When the lift amount is too large, after a certain number of lifts, the cladding surface gradually deviates from the laser focal point, resulting in a significant positive defocus. This prevents effective cladding from continuing on the surface of the previous layer, ultimately causing the actual height of the workpiece to fall far short of the set height. Conversely, when the lift amount is too small, a large negative defocus occurs as cladding progresses, which similarly prevents effective cladding on the surface of the previous cladding layer. Furthermore, this causes excessive melting of the already deposited portion of the workpiece, altering the shape of the formed part and consequently affecting its quality [28,29,30]. As shown in Figure 27, the ideal *Z*-axis lift allows molten metal from zone S_1_ to fill the areas in zones S_2_ and S_3_, resulting in smoother edges of the deposited layer. Based on this, the optimal *Z*-axis lift can be derived.

Assuming that the area of the remelted region *S*_1_ is equal to the sum of the areas of the gap regions *S*_2_ and *S*_3_ on either side [31], obtain:

(10) $$S_{1}=S_{2}+S_{3}$$

From the above equation, derive the relationship between the areas of the semicircular AED and the rectangular ABCD:

(11) $$S_{\mathrm{AED}}=S_{\mathrm{ABCD}}$$

Subtracting the area of triangle OAD from the area of sector OAED gives:

(12) $$S_{\mathrm{AED}}=S_{\mathrm{OAED}}-S_{\mathrm{OAD}}$$

The relationship between the radius *r* of the circle inscribed around the melt cross-section and the melt width *W* and melt height *H* can be derived from Equation (5). Substituting the above expression into Equation (12) yields:

(13) $$S_{\mathrm{AED}}={\left(\frac{4H^{2}+W^{2}}{8H}\right)}^{2}\arcsin(\frac{4WH}{4H^{2}+W^{2}})-\frac{W(W^{2}-4H^{2})}{16H}$$

Based on the area relationships, conclude that regions ABCD:

(14) $$S_{\mathrm{ABCD}}=\Delta Z\cdot W$$

From Equations (13) and (14), the theoretical single-lift increment ΔZ is given by:

(15) $$\Delta Z=\frac{{\left(4H^{2}+W^{2}\right)}^{2}}{64H^{2}W}\arcsin(\frac{4WH}{4H^{2}+W^{2}})-\frac{\left(W^{2}-4H^{2}\right)}{16H}$$

**Figure 28:**
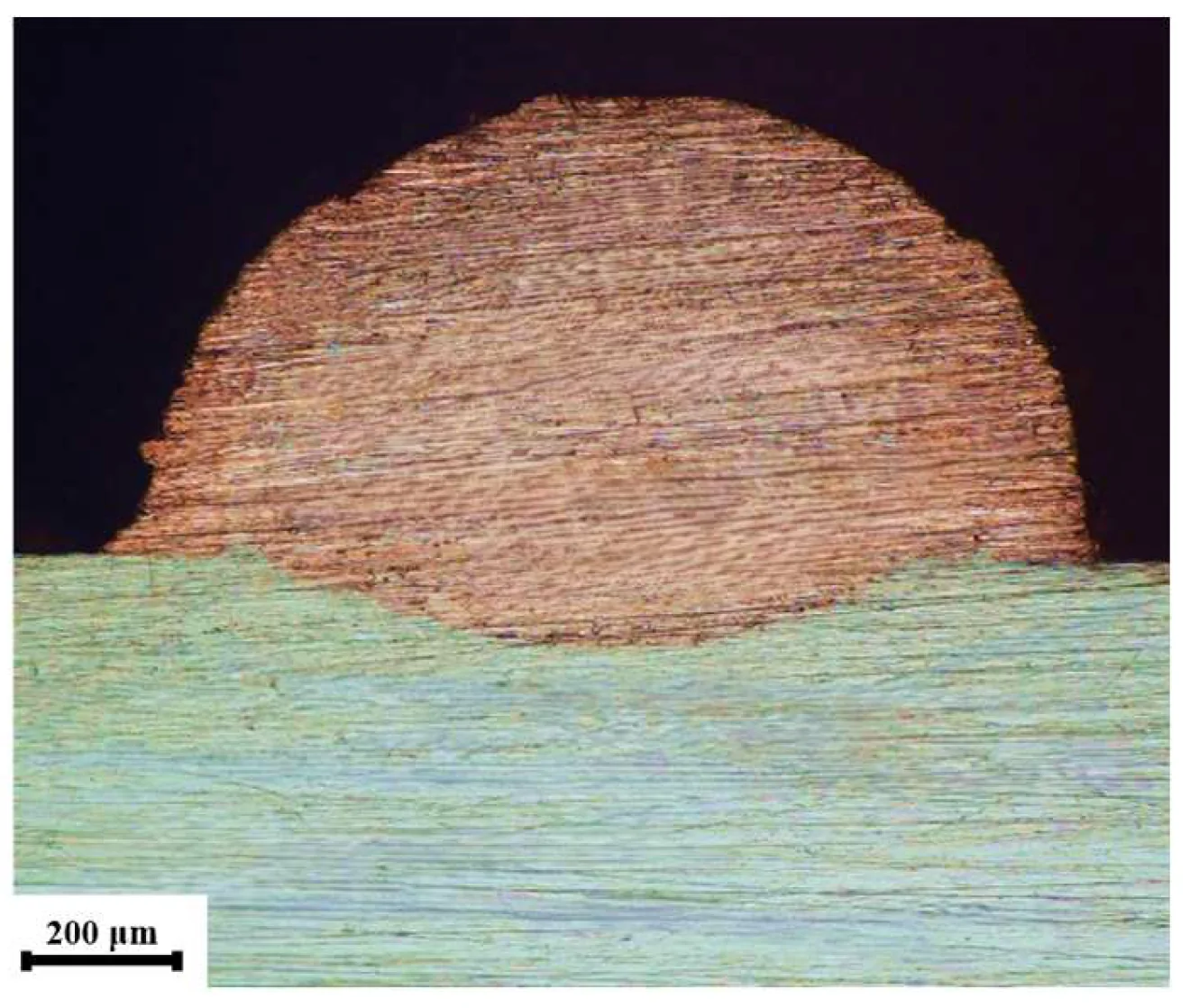
Cross-sectional shape.

Based on the orthogonal test results, the optimized processing condition was selected as a laser power of 500 W, a powder feed rate of 1.1 r/min, and a scanning speed of 800 mm/min. Under this condition, the measured melt width and height were 1.341 mm and 0.613 mm, respectively. By introducing these parameters into the proposed equation, the calculated theoretical increment of Δ*Z* was 0.47 mm. The detailed optimization results are summarized in Table 18, while the corresponding cross-sectional morphology is presented in Figure 28.

## 6. Conclusions

(1) As the laser power *P* increases, the melt width, melt height, and melt depth all increase. As the powder feed rate *F* increases, the melt height increases significantly, the melt depth increases slightly, and the melt width shows no significant change. As the scanning speed *S* increases, the melt height decreases significantly, while the melt width and melt depth decrease slightly. Based on these findings, the following orthogonal experimental parameter ranges were selected: laser power *P* from 400 to 500 W, powder feed rate *F* from 1.1 to 1.3 r/min, and scan speed *S* from 600 to 800 mm/min.

(2) Through range analysis, it was found that the degree of influence of each parameter on the shape coefficient, from greatest to least, was: scan speed *S*, powder feed rate *F*, and laser power *P*. The degree of influence on the dilution ratio, from greatest to least, was: laser power *P*, powder feed rate *F*, and scanning speed *S*. Through analysis of variance for the shape coefficient and dilution ratio, the optimal scheme was determined to be *P*5*F*1*S*5, i.e., laser power of 500 W, powder feed rate of 1.1 r/min, and scanning speed of 800 mm/min.

(3) Combining theoretical models with experimental data, determined that the optimal overlap amount and *Z*-axis lift height are 0.75 mm and 0.47 mm, respectively, and corresponding to an overlap ratio of 44%.

## Figures and Tables

**Table 1:** Parameters of laser.

Name of Parameter	Parameter Values
Model	RFL-C2000S-CE
Average output power (W)	2000
Central wavelength (nm)	1080 ± 5
Maximum modulation frequency (kHz)	5
Output power instability	±1.5%
Type of cooling	water-cooling
Operating temperature (°C)	10–40

**Table 2:** Parameters of powder feeder.

Name of Parameter	Parameter Values
Work unit/single barrel volume	twin-tub/1.5 L
Maximum rotational speed of the powder tray	10 r/min
Carrier gas types	Nitrogen, argon
Flow regulation range	1~20 L/min
Pressure display range	−0.1~1 Mpa
Support powder preheating temperature	<80°
Support powder particle size	74−149 μm (100~200 mesh)
The stirring module supports powder particle size	20−74 μm (200~625 mesh)
Repeat powder feeding accuracy	±3%

**Table 3:** Stainless steel 316L main elements and content.

Elements	Cr	Mn	Mo	Ni	Si	C	Fe
Mass fraction (wt%)	16.5	1.74	2.66	13.1	0.73	0.023	Bal.

**Table 4:** Elemental content of CuCrZr Copper alloy powder.

Elements	Cr	Zr	Cu
Mass fraction (wt%)	0.56	0.21	Bal.

**Table 5:** Experimental scheme and results of laser power single factor.

No.	*P* (W)	*F* (r/min)	*S* (mm/min)	*W* (μm)	*H* (μm)	*h* (μm)	*δ*	*η* (%)
1#	350	1.0	660	1044.74	502.0	42.95	2.08	7.88
2#	400			1044.90	512.6	72.83	2.04	12.44
3#	450			1200.00	541.9	38.67	2.21	6.66
4#	500			1230.06	537.4	69.72	2.29	11.48
5#	550			1233.82	530.3	71.05	2.33	11.82
6#	600			1193.96	532.0	91.84	2.24	14.72
7#	650			1266.75	542.4	79.71	2.34	12.81
8#	700			1457.11	533.4	113.84	2.73	17.59

**Table 6:** Single-factor experimental scheme and results of powder feeding rate.

No.	*P* (W)	*F* (r/min)	*S* (mm/min)	*W* (μm)	*H* (μm)	*h* (μm)	*δ*	*η* (%)
1#	500	0.8	660	1200.89	386.43	83.18	3.11	17.71
2#		0.9		1256.35	481.74	81.45	2.61	14.46
3#		1.0		1228.64	478.33	84.91	2.57	15.08
4#		1.1		1317.01	616.92	100.51	2.13	14.01
5#		1.2		1296.20	655.09	105.71	1.98	13.89
6#		1.3		1287.98	712.29	113.84	1.81	13.78
7#		1.4		1423.93	757.49	107.28	1.88	12.41
8#		1.5		1268.45	787.10	68.30	1.61	7.98

**Table 7:** Single-factor experimental scheme and results of scanning speed.

No.	*P* (W)	*F* (r/min)	*S* (mm/min)	*W* (μm)	*H* (μm)	*h* (μm)	*δ*	*η* (%)
1#	500	1.3	480	1496.81	946.68	93.14	1.58	8.96
2#			540	1293.20	844.79	86.67	1.53	9.30
3#			600	1431.83	779.91	108.31	1.84	12.19
4#			660	1371.18	706.17	116.99	1.94	14.21
5#			720	1282.35	630.37	129.97	2.03	17.09
6#			780	1358.17	606.51	125.63	2.24	17.16
7#			840	1189.20	569.69	64.98	2.09	10.24
8#			900	1150.21	545.86	71.48	2.11	11.58

**Table 8:** Orthogonal experimental factors and levels.

	Factors	Laser Power *P* (W)	Powder Feed Rate *F* (r/min)	Scan Speed *S* (mm/min)
Levels				
Level 1		400	1.1	600
Level 2		425	1.15	650
Level 3		450	1.2	700
Level 4		475	1.25	750
Level 5		500	1.3	800

**Table 9:** Orthogonal experimental scheme and results.

No.	*P* (W)	*F* (r/min)	*S* (mm/min)	*W* (μm)	*H* (μm)	*h* (μm)	*δ*	*η* (%)
1#	400	1.1	600	1231.71	711.82	81.98	1.73	10.33
2#	425	1.1	650	1231.68	667.83	57.99	1.84	7.99
3#	450	1.1	700	1183.70	649.83	65.98	1.82	9.22
4#	475	1.1	750	1339.66	603.85	127.97	2.22	17.49
5#	500	1.1	800	1341.75	613.90	119.99	2.19	16.35
6#	400	1.15	600	1087.72	675.83	55.99	1.61	7.65
7#	425	1.15	650	1287.67	677.83	74.01	1.90	9.84
8#	450	1.15	700	1191.70	619.84	59.99	1.92	8.82
9#	475	1.15	750	1325.66	575.85	93.98	2.30	14.03
10#	500	1.15	800	1371.65	723.82	51.99	1.90	6.70
11#	400	1.2	700	1197.80	691.90	41.99	1.73	5.72
12#	425	1.2	750	1203.73	621.92	59.99	1.94	8.80
13#	450	1.2	800	1287.80	637.92	98.00	2.02	13.32
14#	475	1.2	600	1325.76	777.84	76.01	1.70	8.90
15#	500	1.2	650	1443.77	713.92	145.98	2.02	16.98
16#	400	1.25	750	1077.84	647.88	31.99	1.66	4.71
17#	425	1.25	800	1141.74	647.86	37.99	1.76	5.54
18#	450	1.25	600	1311.82	787.86	117.99	1.67	13.03
19#	475	1.25	650	1433.68	797.82	101.97	1.80	11.33
20#	500	1.25	700	1417.75	739.91	109.99	1.92	12.94
21#	400	1.3	800	1293.71	619.85	35.99	2.09	5.49
22#	425	1.3	600	1401.81	873.86	103.99	1.60	10.63
23#	450	1.3	650	1303.69	833.83	27.99	1.56	3.25
24#	475	1.3	700	1319.72	779.86	65.98	1.69	7.80
25#	500	1.3	750	1271.68	707.82	93.98	1.80	11.72

**Table 10:** Extreme difference analysis of shape factor.

	Factors	Laser Power *P* (W)	Powder Feed Rate *F* (r/min)	Scan Speed *S* (mm/min)
Average				
K1		1.76	1.96	1.66
K2		1.81	1.93	1.82
K3		1.80	1.88	1.82
K4		1.94	1.76	1.98
K5		1.96	1.75	1.99
R		0.20	0.21	0.33
Impact level		3	2	1
Excellent Level		500	1.1	800
Optimal Combination		*P*5*F*1*S*5		

**Table 11:** Extreme difference analysis of dilution rate.

	Factors	Laser Power *P* (W)	Powder Feed Rate *F* (r/min)	Scan Speed *S* (mm/min)
Average				
K1		6.78	12.27	9.92
K2		8.56	9.41	9.44
K3		9.53	10.74	9.11
K4		11.91	9.51	10.31
K5		12.94	7.78	10.94
R		6.16	4.50	1.84
Impact level		1	2	3
Excellent Level		500	1.1	800
Optimal Combination		*P*5*F*1*S*5		

**Table 12:** Relationship between *p*-value and significance.

*p* Value	Significance
*p* < 0.001	***
0.001 ≤ *p* < 0.01	**
0.01 ≤ *p* < 0.05	*
*p* > 0.05	-

*** Extremely significant, ** Significant, * Statistically significant, - Without significance.

**Table 13:** ANOVA of shape coefficients.

Source	Degrees of Freedom	Adj SS	Adj MS	F Value	*p* Value	Contribution Rate (%)	Significance
*P*	4	0.168	0.042	2.21	0.124	17.6	-
*F*	4	0.184	0.046	2.42	0.095	19.2	-
*S*	4	0.376	0.094	4.95	0.011	39.3	*
Error	12	0.228	0.019				
Total	24	0.956					

- Without significance, * Statistically significant.

**Table 14:** Shape coefficient values.

	*F* (r/min)	1.1	1.15	1.2	1.25	1.3
*S* (mm/min)						
600		1.73	1.61	1.70	1.67	1.60
650		1.84	1.90	2.02	1.80	1.56
700		1.82	1.92	1.73	1.92	1.69
750		2.22	2.30	1.94	1.66	1.80
800		2.19	1.90	2.02	1.76	2.09

**Table 15:** ANOVA of dilution rate.

Source	Degrees of Freedom	Adj SS	Adj MS	F Value	*p* Value	Contribution Rate (%)	Significance
*P*	4	124.69	31.17	2.25	0.124	34.89	-
*F*	4	56.15	14.04	1.01	0.438	15.71	-
*S*	4	10.46	2.61	0.19	0.940	2.93	-
Error	12	166.07	13.84				
Total	24	357.38					

- Without significance.

**Table 16:** Dilution rate of different process conditions.

	*F* (r/min)	1.1	1.15	1.2	1.25	1.3
*P* (W)						
400		10.33	7.65	5.72	4.71	5.49
425		7.99	9.84	8.80	5.54	10.63
450		9.22	8.82	13.32	13.03	3.25
475		17.49	14.03	8.90	11.33	7.80
500		16.35	6.70	16.98	12.94	11.72

**Table 17:** Experimental design for transverse overlap ratio.

No.	*P* (W)	*F* (r/min)	*S* (mm/min)	Overlap Ratio *λ* (%)	Center-to-Center Distance *d* (mm)
1#	500	1.1	800	47.5	0.7
2#				44	0.75
3#				40.5	0.8
4#				33	0.9
5#				23	1.03
6#				21	1.06

**Table 18:** Results of parameter optimization.

Parameters	Value
Laser Power (W)	500
Powder Feed Rate (r/min)	1.1
Scanning Speed (mm/min)	800
Melt Width (μm)	1341.75
Melt Height (μm)	613.90
Melt Depth (μm)	119.99
Shape Factor	2.19
Dilution Ratio (%)	16.35
Overlap Ratio (%)	44
*Z*-Axis Lift (mm)	0.47

## Data Availability

The original contributions presented in this study are included in the article. Further inquiries can be directed to the corresponding author.
